# The association between air pollutants, meteorological factors, and mushroom poisoning cases in Guizhou Province, China: a 5-year time-series study

**DOI:** 10.3389/fpubh.2025.1699557

**Published:** 2026-01-08

**Authors:** Muli Wu, Anzhong Wu, Qingyuan Chen, Sufang Xiong, Yanrong Hu, Shuai Huang, Qing Wang, Jun Li, Hua Guo

**Affiliations:** 1 Institute of Public Health Surveillance and Evaluation, Guizhou Center for Disease Control and Prevention, Guiyang, China; 2 School of Public Health, The Key Laboratory of Environmental Pollution Monitoring and Disease Control, Ministry of Education, Guizhou Medical University, Guiyang, China; 3 Experimental Center, Guizhou Center for Disease Control and Prevention, Guiyang, China

**Keywords:** mushroom poisoning, air pollutant, meteorological factor, distributed lag nonlinear model, time-series study

## Abstract

**Background:**

Mushroom poisoning is a major food safety concern in Guizhou Province, where meteorological conditions play an important role in its occurrence. However, the lagged associations and interactions between air pollutants and meteorological factors on poisoning risk remain unclear. This study systematically evaluates the impact of these environmental factors on mushroom poisoning incidence.

**Methods:**

We collected daily records of mushroom poisoning cases, air pollutants, and meteorological data in Guizhou Province from 2019 to 2023. A generalized additive model (GAM) was employed to analyze exposure-response relationships and interactions between air pollutants, meteorological factors, and mushroom poisoning cases, while a distributed lag nonlinear model (DLNM) quantified lagged associations.

**Results:**

In 2019 and 2023, Guizhou Province reported 5,927 mushroom poisoning cases, with rural areas accounting for 4,306 cases (72.7%) and urban areas for 1,621 (27.3%). Significant nonlinear relationships were observed between the risk of mushroom poisoning and air pollutants [CO, O_3_, SO_2,_ Particulate Matter 2.5(PM_2.5_)] and meteorological factors [rainfall (RF), relative humidity (RH), sunshine duration (SSD), daily average 5 cm ground temperature (T5)]. Single-pollutant DLNM analysis demonstrated that 0.1 mg/m^3^ increases in CO reduced mushroom poisoning risk (RCO = 0.68, 95% CI: 0.47–0.97, lag 0–18 days), as did 10 μg/m^3^ increases in O_3_ and PM_2.5_ (RR_O3_ = 0.75, 95% CI: 0.66–0.86, lag 0–20 days; RR_PM2.5_ = 0.28, 95% CI: 0.20–0.38, lag 0–20 days). In contrast, single-factor DLNM meteorological model analysis identified higher T5 (P_75_: 26 °C and P_97.5_: 30 °C; RRP_75_ = 5.53, 95% CI: 2.45–12.47, lag 0–20; RR_97.5_ = 10.32, 95% CI: 2.89–36.83, lag 0–20), RF (P_75_: 4 mm; RR = 3.21, 95% CI: 1.31–7.86, lag 0–17), and RH (P_75_: 87%; RR = 2.56, 95% CI: 1.05–76.23, lag 0–20) as risk factors for mushroom poisoning. Moreover, signification interactions between meteorological factors and air pollutants amplified the risk of mushroom poisoning.

**Conclusion:**

This study revealed lagged associations and interactions between air pollutants and meteorological factors on mushroom poisoning, providing a scientific basis for precise prevention and control.

## 1 Introduction

Mushrooms are fleshy fungi that develop from mycelium and are broadly classified as edible or poisonous (1). Despite the widespread consumption of edible mushrooms, poisoning due to the accidental ingestion of poisonous mushrooms has become a major global public health challenge (2–4). Mushroom poisoning can lead to gastroenteritis, neurological disorders, liver failure, and other symptoms, which can be life-threatening (5–7). In recent years, the frequency of foodborne diseases has been increasing, especially in low-resource countries and regions (8). In China, mushroom poisoning is the leading cause of foodborne disease outbreaks and is associated with a notable fatality rate (9). From 2010 to 2020, 10,036 mushroom poisoning outbreaks were reported in China, involving 38,676 cases and 788 deaths (10). The southwestern and central regions exhibit particularly high incidence rates and outbreak frequencies (11). Therefore, mushroom poisoning urgently requires urgent attention in prevention and improved control strategies.

Guizhou province, a typical high-incidence area, has a warm and humid climate, abundant precipitation, and dense vegetation (12), which provides favorable conditions for mushroom growth. From 2011 to 2023, Guizhou province reported 2,518 mushroom poisoning outbreaks, ranking third nationwide, with 8,716 cases and 97 deaths. In addition, the annual number of cases is increasing (13, 14).

The occurrence of mushroom poisoning is closely related to multiple interacting factors, including environmental, social, and economic conditions (15, 16). However, existing studies have mainly focused on socioeconomic factors (7, 17, 18), while the exploration of key environmental drivers is lacking. With the intensification of climate change, the impacts of meteorological conditions and air pollutants on public health have become major research hotspots (19–24). In the field of foodborne diseases, most studies have focused on bacterial diseases, demonstrating that temperature extremes, precipitation, and their interactions significantly affect the incidence of bacterial foodborne diseases (8, 25). In addition, studies have revealed the lagged and cumulative association of environmental exposures using distributional lag nonlinear modeling (DLNM) (26, 27).

Compared with bacterial foodborne diseases, studies on the relationship between environmental factors and mushroom poisoning remain scarce and fragmented. Some studies have reported potential associations between monthly rainfall or mean air temperature and the number of poisoning cases (15, 28, 29) and identified a nonlinear exposure-response relationship between meteorological factors and mushroom poisoning (30). Nevertheless, a systematic theoretical framework has yet to be developed. In particular, air pollutants, as key environmental stressors, have not yet been thoroughly investigated regarding their independent influences on mushroom poisoning risk, interactive associations with meteorological factors, and lag and cumulative lag associations. This knowledge gap limits the foresight and precision of existing public health prevention and control strategies.

To address this, we selected Guizhou Province, a high-incidence area, as the study site. Using a combination of a generalized additive model (GAM) and DLNM, this study systematically assessed the associations between air pollutants and meteorological factors in relation to the risk of developing mushroom poisoning. The integration of air pollutants and meteorological factors at the provincial scale represents a core innovation, aiming to reveal their independent associations, interactive associations, and lagged and cumulative lagged associations. The results provide a solid scientific basis for building a precise and efficient early warning, prevention, and control system for mushroom poisoning and offer an important reference for managing environmental health risks in similar regions worldwide.

## 2 Materials and methods

### 2.1 Study area

Guizhou Province is located in the southeastern part of China’s southwest region, ranging from 103°36′E to 109°35′E and 24°37′N to 29°13′N. It covers a total area of about 176,000 km^2^, with a total of nine municipalities (states), and has a warm and humid climate (12). At the end of 2024, the resident population of Guizhou Province was 38.65 million people. The geographical location of the study area is shown in Supplementary Figure S1.

### 2.2 Data sources

Mushroom poisoning outbreak data from 1 January 2019 to 31 December 2023 were obtained from the Foodborne Disease Outbreak Surveillance System of Guizhou Province. Map data were obtained from the National Geographic Information Public Service Platform.^1^ Daily average air pollutant concentrations were obtained from the National Urban Air Quality Real-Time Distribution Platform.^2^ The air pollutants considered in this study were Particulate Matter 2.5 (PM_2.5_; μg/m^3^), Particulate Matter 10 (PM_10_; μg/m^3^), CO (mg/m^3^), NO_2_ (μg/m^3^), SO_2_ (μg/m^3^), and O_3_ (μg/m^3^). Daily meteorological data were obtained from the Guizhou Provincial Meteorological Bureau,^3^ including average daily air temperature (AT; °C), daily average 5 cm ground temperature (T5; °C), daily rainfall (RF; mm), relative humidity (RH; %), average wind speed (AW; m/s), and sunshine duration (SSD; h). Data integrity is high, and missing values are minimal. For the few missing data points, the average of the monitoring values of the 2 days before and after was used to fill in the data to ensure the continuity and reliability of the data; the daily data for air pollutants and meteorological factors were first obtained by calculating the daily average values of each urban area in Guizhou Province, and then the daily average of the whole Guizhou Province was further calculated based on the daily mean values for each city area.

### 2.3 Statistical analysis

#### 2.3.1 Descriptive analysis

Means, standard deviations, and quartiles were used to describe the distribution of air pollutants, meteorological factors, and the number of cases of mushroom poisoning during the study period and to plot the time series of the number of cases of air pollutants, meteorological factors, and mushroom poisoning for the years 2019–2023.

#### 2.3.2 Spearman correlation analysis

Spearman correlation analysis was used to explore the correlation between air pollutants and meteorological factors. When the correlation coefficient between variables exceeds 0.7, it is considered that there is a strong correlation, and these highly correlated variables should be excluded as needed during the model fitting process to avoid potential covariance problems (31).

#### 2.3.3 Construction of statistical models

To comprehensively resolve the complex relationship between environmental factors and the number of mushroom poisoning episodes, the GAM and DLNM frameworks were jointly adopted. GAM was chosen over the parametric generalized linear model (GLM) because GAM can flexibly capture the potential nonlinear relationship between environmental exposures and health outcomes through a nonparametric smoothing function. In contrast, the linear or simple parametric form presupposed by GLM may not be able to adequately fit such complex biological responses (30, 32). The DLNM was introduced as it can simultaneously characterize the “response dimension” and “lag dimension” of environmental exposure within a unified framework (32). The specific analysis strategies were as follows: (1) explore the nonlinear exposure-response relationship between air pollutants and meteorological factors on mushroom poisoning using GAM and test the interaction associations; (2) construct the cross-basis function within the DLNM framework to accurately quantify the lagged association of single-day exposure on the risk of morbidity and its cumulative health associations within a specific time window. Given that the number of mushroom poisoning cases was count data and over-dispersed, all models were fitted based on quasi-Poisson regression to correct for the fact that the variance was larger than the mean. The definition of GAM is detailed in Equation 1:


$$\log[E(Y_{t})]=\alpha +\sum_{i=1}^{k}s(X_{i},\mathrm{df})+s(\text{time},\mathrm{df})+\text{holiday}+\mathrm{DOW}$$ (1)

where *Y_t_* is the number of mushroom poisoning cases on day t; *E(Y_t_)* is the expected value of the number of mushroom poisoning cases on day t; *α* is the intercept; *s()* is the smoothing spline function; *X_i_* is the independent variables (meteorological factors, air pollutants during the same period); *time* is the variable used to control the long-term time trend; *df* is the degree of freedom; *holiday* is the dummy variable for the holiday association; and *DOW* is the dummy variable for the day of week association. According to the minimum Akaike information criterion (AIC), the degrees of freedom for time in the model were set to 7, while those for meteorological factors and atmospheric pollutants were set to 3.

The definition of DLNM is detailed in Equation 2.


$$Y_{t}\sim \text{Quasi}-\text{Poission}(Y_{t})$$

$$\begin{array}{c} \log(Y_{t})=\alpha +\mathrm{cb}(M,\mathrm{lag},\mathrm{df})+\mathrm{ns}(\text{Weather},\mathrm{df})\\ +\mathrm{ns}(\text{Pollutants},\mathrm{df})+\mathrm{ns}(\text{time},\mathrm{df})+\text{holiday}+\mathrm{DOW} \end{array}$$ (2)

where *cb()* is the cross-basis function established by the study variables and lag time; *M* is the meteorological factor or air pollutant variable studied; lag is the maximum lag time set; *ns()* is the natural spline function; *Weather* and *Pollutants* are the meteorological factor or air pollutant variables other than the study factors, used to control for confounding associations; and *α*, *Yt*, *df*, *time*, *holiday,* and *DOW* have the same meaning as in GAM. According to the minimum AIC, the range of degrees of freedom for the time in the model was set to 7–9, while the degrees of freedom for meteorological factors and air pollutants were set to 2 or 3 in both the exposure-response dimension and exposure-lag dimension. Since no previous studies have investigated the lagged relationship between mushroom poisoning and environmental factors, we referred to studies on the growth cycle of mushrooms, as well as the environmental correlation of other diseases. Based on sensitivity analyses of the established model, the maximum lag time was determined to be 20 days (18, 30, 33, 34). We constructed both one-factor and multi-factor models for meteorological factors and air pollutants, respectively, and conducted sensitivity analyses by varying model parameters and the maximum lag time.

We used the median of the study factors as a reference (26) to calculate the change in relative risk (RR) and cumulative relative risk (CRR) of mushroom poisoning for a unit increase in the concentration of each pollutant. Additionally, we calculated the RR and CRR of each meteorological factor at different lag days and across various percentiles (P_2.5_, P_25_, P_75_, P_97.5_) to systematically assess the lag association and cumulative lag association of air pollutants and meteorological factors on the risk of mushroom poisoning. ArcGIS 10.8 was used for map drawing, and all statistical analyses and modeling were carried out in R 4.4.2 with the significance level set at 0.05.

## 3 Results

### 3.1 Characteristics of air pollutants, meteorological factors, and mushroom poisoning cases

**Figure 1:**
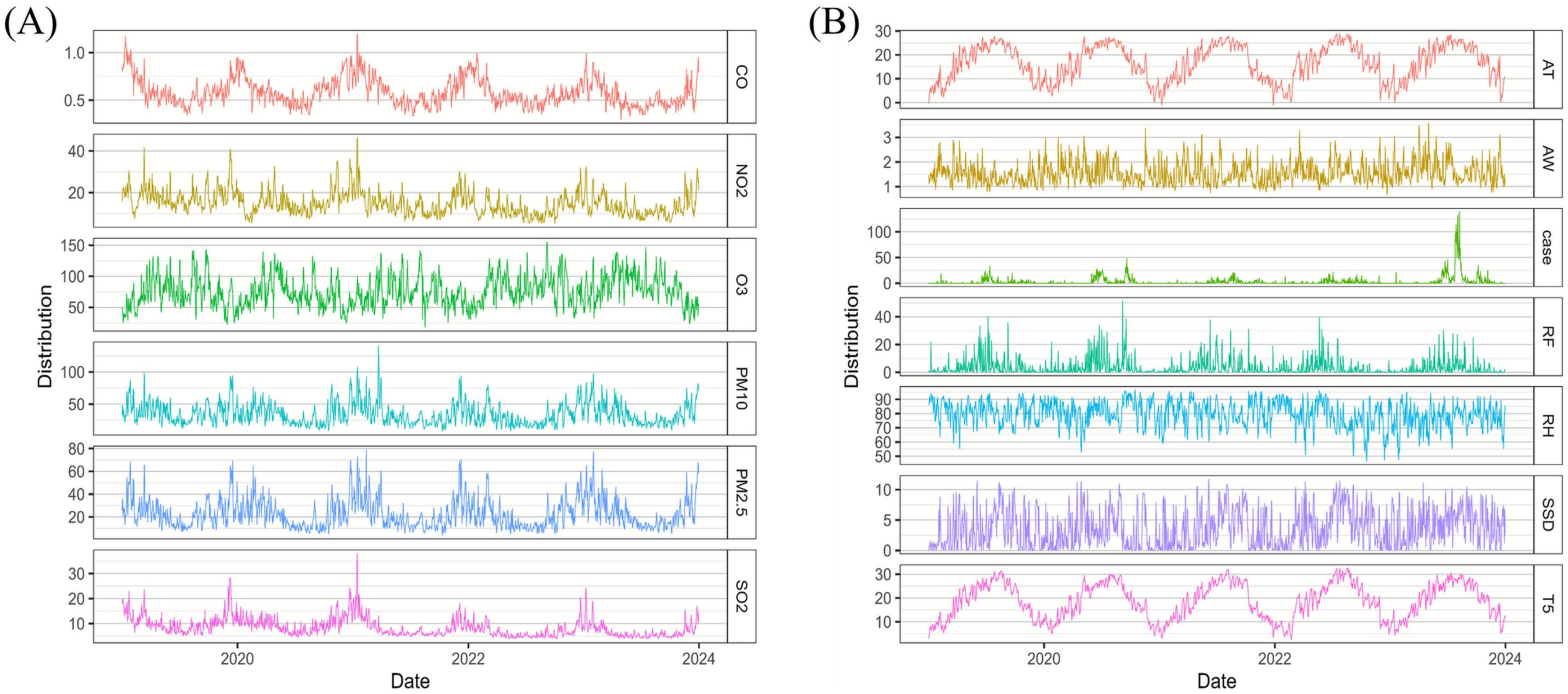
Time series plot of air pollutants **(A)**, meteorological factors, and the number of mushroom poisoning cases **(B)**. AT, average daily temperature; AW, average wind speed; Case, number of cases of mushroom poisoning; RF, rainfall; RH, relative humidity; SSD, sunshine duration; T5, daily average 5 cm ground temperature; PM_2.5_, Particulate Matter 2.5; PM_10_, Particulate Matter 10.

From 2019 to 2023, a total of 5,927 cases of mushroom poisoning were reported in Guizhou Province, including 4,306 cases (72.7%) in rural areas and 1,621 cases (27.3%) in urban areas. The daily distribution of air pollutants, meteorological factors, and the number of mushroom poisoning cases were cyclical (Figure 1). The daily average values for PM_10_, PM_2.5_, SO_2_, NO_2_, CO, O_3_, AT, T5, AW, RF, RH, SSD, and the number of mushroom poisoning cases are shown in Table 1 below. AW was not included in the follow-up analyses because of its low correlation with the number of mushroom poisoning cases. AT, PM_10_, and NO_2_ were also excluded to avoid multicollinearity (Supplementary Table S1).

**Table 1:** Distribution characteristics of air pollutants, meteorological factors and the number of mushroom poisoning cases in Guizhou Province from 2019 to 2023.

Variable	Mean ± SD	Minimum	P_25_	Median	P_75_	Maximum
Total cases	3.25 ± 9.99	0.00	0.00	0.00	3.00	139.00
Rural cases	2.36 ± 7.61	0.00	0.00	0.00	2.00	122.00
City cases	0.89 ± 3.20	0.00	0.00	0.00	0.00	46.00
AT	16.61 ± 7.40	−1.06	10.35	17.24	23.40	28.88
RF	3.29 ± 5.65	0.00	0.13	0.87	3.90	51.43
RH	79.58 ± 8.91	46.38	73.50	80.40	86.51	96.62
SSD	3.64 ± 3.09	0.00	0.82	2.97	6.09	11.76
AW	1.56 ± 0.45	0.69	1.23	1.46	1.80	3.60
T5	18.73 ± 7.51	2.51	12.12	19.18	25.59	32.61
PM_2.5_	22.73 ± 12.76	4.22	12.89	19.56	29.78	78.79
PM_10_	34.53 ± 17.21	8.22	21.44	30.45	43.67	140.22
SO_2_	8.46 ± 3.81	3.89	5.67	7.44	10.11	37.89
NO_2_	14.92 ± 5.69	5.33	10.78	14.00	18.11	46.11
O_3_	75.67 ± 24.60	18.11	58.78	72.33	91.67	155.00
CO	0.58 ± 0.14	0.30	0.48	0.55	0.66	1.19

SD, standard deviation; P_x_, xth percentile; AT, average daily temperature; AW, average wind speed; Case, number of cases of mushroom poisoning; RF, rainfall; RH, relative humidity; SSD, sunshine duration; T5, daily average 5 cm ground temperature; PM_2.5_, Particulate Matter 2.5; PM_10_, Particulate Matter10.

### 3.2 Association between air pollutants, meteorological factors, and mushroom poisoning

**Figure 2:**
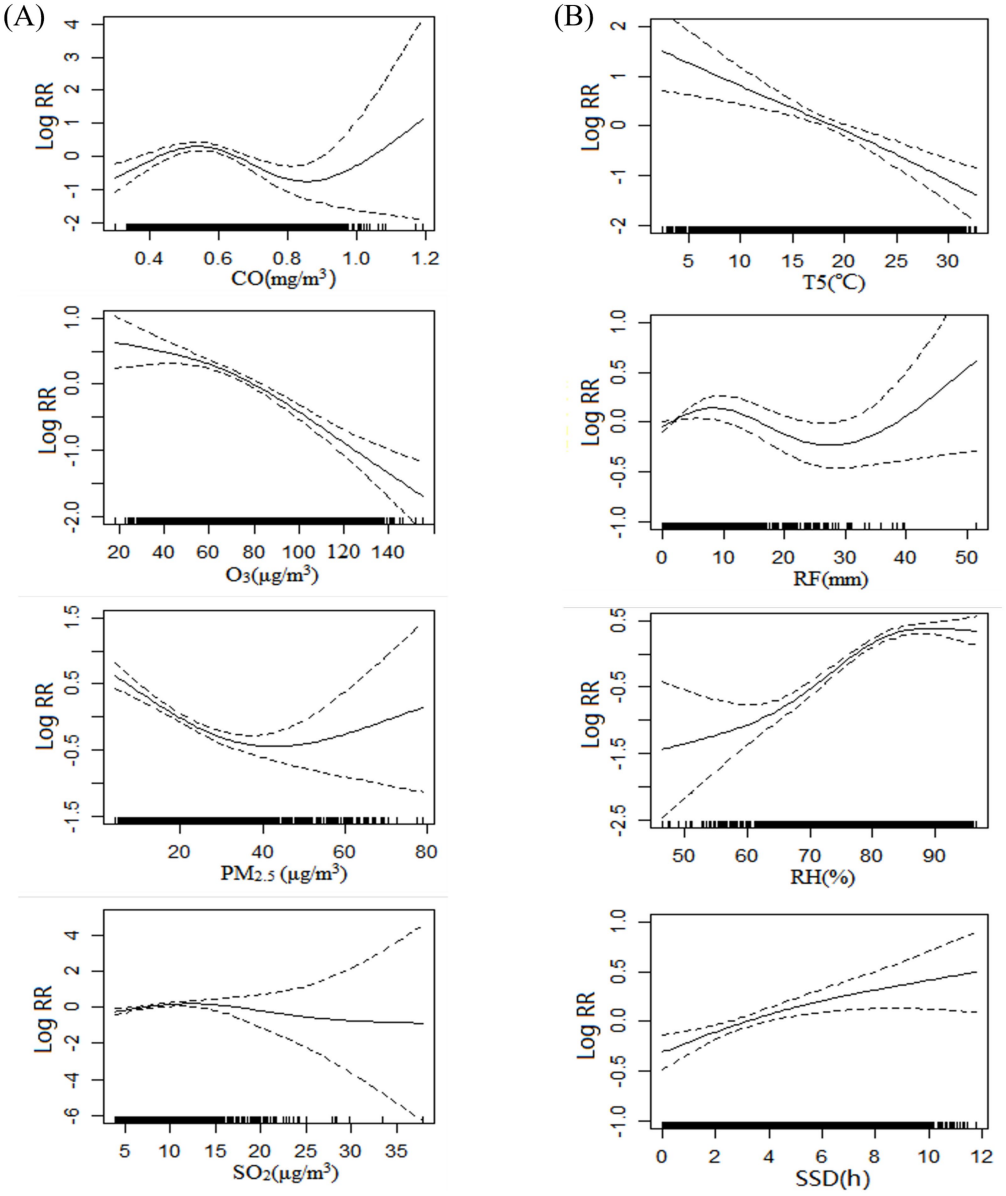
Relationship between the number of mushroom poisoning cases and air pollutants **(A)** (CO, O_3_, PM_2.5_, SO_2_) and meteorological factors **(B)** (T5, RF, RH, SSD). The solid lines indicate the logarithmic relative risk of mushroom poisoning, and the dotted lines indicate the 95% confidence level. RF, rainfall; RH, relative humidity; SSD, sunshine duration; T5, daily average 5 cm ground temperature; PM_2.5_, particulate matter 2.5.

The exposure-response relationship between air pollutants/meteorological elements and the number of mushroom poisoning cases was preliminarily investigated using a GAM. The analysis showed that variations in the concentrations of air pollutants (CO, O_3_, PM_2.5_, SO_2_) and mushroom poisoning had a significant nonlinear relationship (Figure 2A). Similarly, variations in meteorological parameters (T5, RF, RH, SSD) showed a nonlinear relationship with the risk of poisoning (Figure 2B). Based on these findings, we subsequently developed a DLNM for in-depth exploration.

### 3.3 Lag association of air pollutants on the risk of mushroom poisoning

#### 3.3.1 Air pollutants and the total number of cases of mushroom poisoning

**Figure 3:**
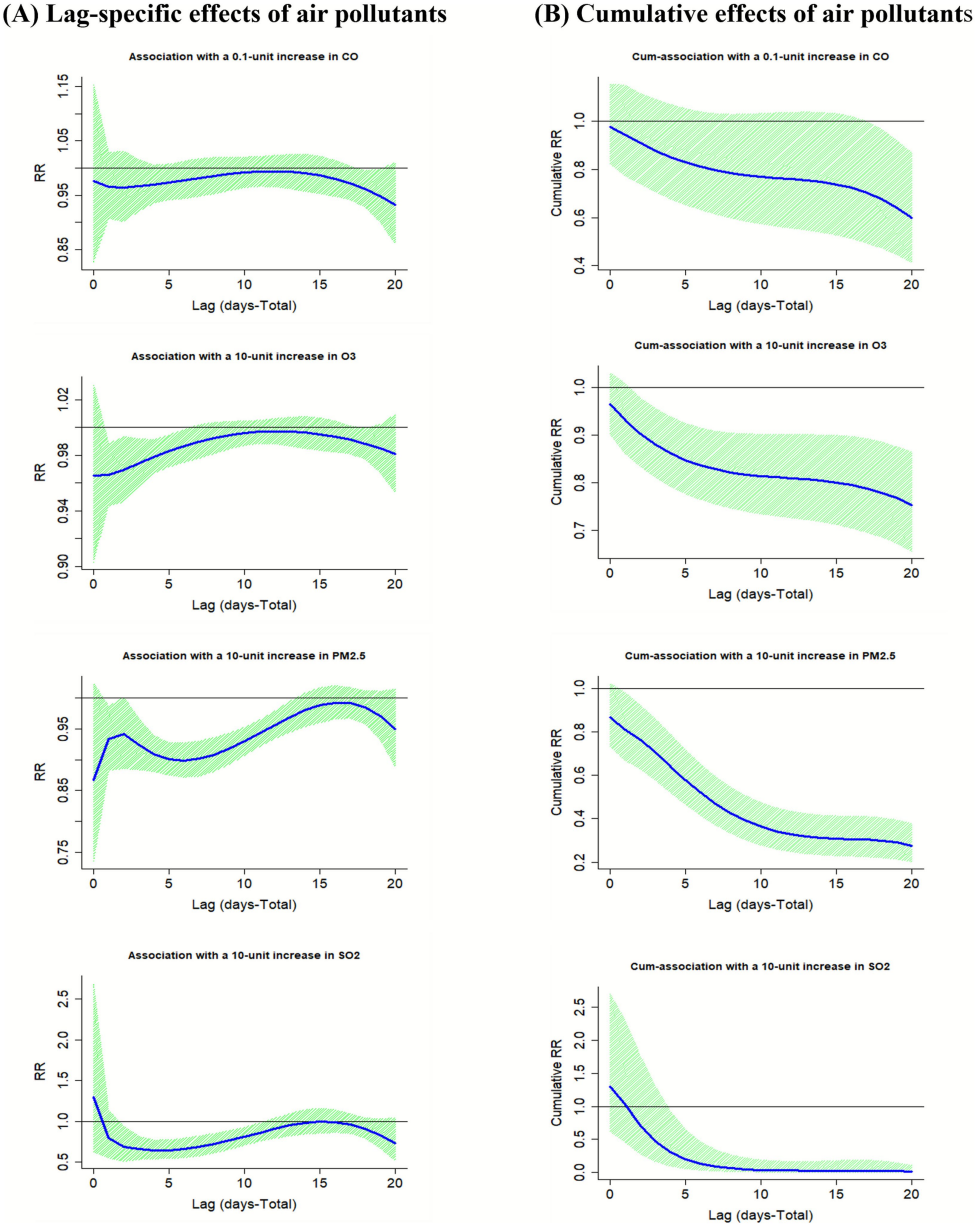
Association of air pollutants (CO, O_3_, PM_2.5_, SO_2_) on the risk of mushroom poisoning at specific **(A)** and cumulative **(B)** lag times. Single-factor model was adjusted for T5, RF, RH, and SSD. RF, rainfall; RH, relative humidity; SSD, sunshine duration; T5, daily average 5 cm ground temperature; PM_2.5_, particulate matter 2.5.

Figure 3 and Supplementary Tables S1–S4 illustrate the association between air pollutants (CO, O_3_, PM_2.5_, SO_2_) and the risk of mushroom poisoning in the single meteorological factor DLNM model. There was no statistically significant association of a 0.1 mg/m^3^ increase in CO concentration with the risk of mushroom poisoning. However, a significant reduction in cumulative risk was observed at lag 0–18 (RR = 0.68, 95% CI: 0.47–0.97). A 10 μg/m^3^ increase in O_3_ concentration was consistently associated with a reduced risk of mushroom poisoning. Risk was significantly reduced from lag 1 (RR = 0.97, 95% CI: 0.94–0.99) to lag 5 (RR = 0.75, 95% CI: 0.97–0.99). In the cumulative lag analysis, the risk decreased significantly from lag 0–2 (RR = 0.90, 95% CI: 0.83–0.98) to lag 0–20 (RR = 0.75, 95% CI: 0.66–0.86). A 10 μg/m^3^ increase in PM_2.5_ concentration led to a statistically significant reduction in the risk of mushroom poisoning, from lag 3 (RR = 0.92, 95% CI: 0.88–0.97) to lag 13 (RR = 0.97, 95% CI: 0.94–0.99). The cumulative risk also decreased consistently from lag 0–1 (RR = 0.81, 95% CI: 0.67–0.98) to lag 0–20 (RR = 0.28, 95% CI: 0.20–0.38). Similarly, a 10 μg/m^3^ increase in SO_2_ concentration was associated with a reduced risk of mushroom poisoning, from lag 2 (RR = 0.69, 95% CI: 0.51–0.94) to lag 11 (RR = 0.86, 95% CI: 0.75–0.98). The cumulative risk showed a significant decline from lag 0–4 (RR = 0.30, 95% CI: 0.10–0.92) to lag 0–20 (RR = 0.02, 95% CI: 0.00–0.01). The results of the multi-pollutant model analyses were generally consistent with the single-pollutant model (Supplementary Figure S2).

#### 3.3.2 Air pollutants and the number of cases of mushroom poisoning in rural–urban areas

Supplementary Figures S3, S4 show the single-day lag associations and cumulative lag associations of CO, O_3_, PM_2.5_, and SO_2_ on the risk of mushroom poisoning occurrence in urban and rural areas over the 0–20-day lag period. The results showed that in the single-pollutant model, each 0.1 mg/m^3^ increase in CO concentration had no significant association in rural areas, but showed a protective association in urban areas, with a single-day lag association seen from lag 12–lag 17 (RR_mean_ = 0.96, 95% CI: 0.93–0.99), but no cumulative association. Each 10 μg/m^3^ increase in O_3_ concentration showed a significant protective association in rural areas, with a single-day association of lag 1–lag 5 (RR_mean_ = 0.97, 95% CI: 0.96–0.99) and a significant reduction in cumulative risk from lag 0–1 to lag 0–20 (RR_mean_ = 0.81, 95% CI: 0.74–0.90), while only a cumulative risk reduction was observed in urban areas (lag 0–0 to lag 0–20, RR_mean_ = 0.85, 95% CI: 0.75–0.97). Each 10 μg/m^3^ increase in PM_2.5_ concentrations showed significant protective associations in both urban and rural areas: single-day lag association was seen from lag 1–lag 12 in rural areas (RR_mean_ = 0.92, 95% CI: 0.89–0.95), and cumulative associations from lag 0–1 to lag 0–20 (RR_mean_ = 0.45, 95% CI: 0.37–0.57). In urban areas, single-day lag association was observed from lag 4–lag 10 (RR_mean_ = 0.95, 95% CI: 0.92–0.98), and cumulative associations were seen from lag 0–5 to lag 0–20 (RR_mean_ = 0.63, 95% CI: 0.46–0.87). Each 10 μg/m^3^ increase in SO_2_ concentration significantly reduced the risk in rural areas, with a single-day association from lag 1–lag 12 (RR_mean_ = 0.64, 95% CI: 0.54–0.78), and a cumulative association from lag 0–1 to lag 0–5 (RR_mean_ = 0.13, 95% CI: 0.05–0.34), and only a cumulative association in urban areas (lag 0–14 to lag 0–19, RR_mean_ = 0.11, 95% CI: 0.01–0.86). The analysis results of the multi-pollutant model were basically consistent with those of the single-pollutant model (Supplementary Figure S5).

### 3.4 Lag association of meteorological factors on the risk of mushroom poisoning

#### 3.4.1 Lag association of meteorological factors on the risk of mushroom poisoning during 0–20 days

**Figure 4:**
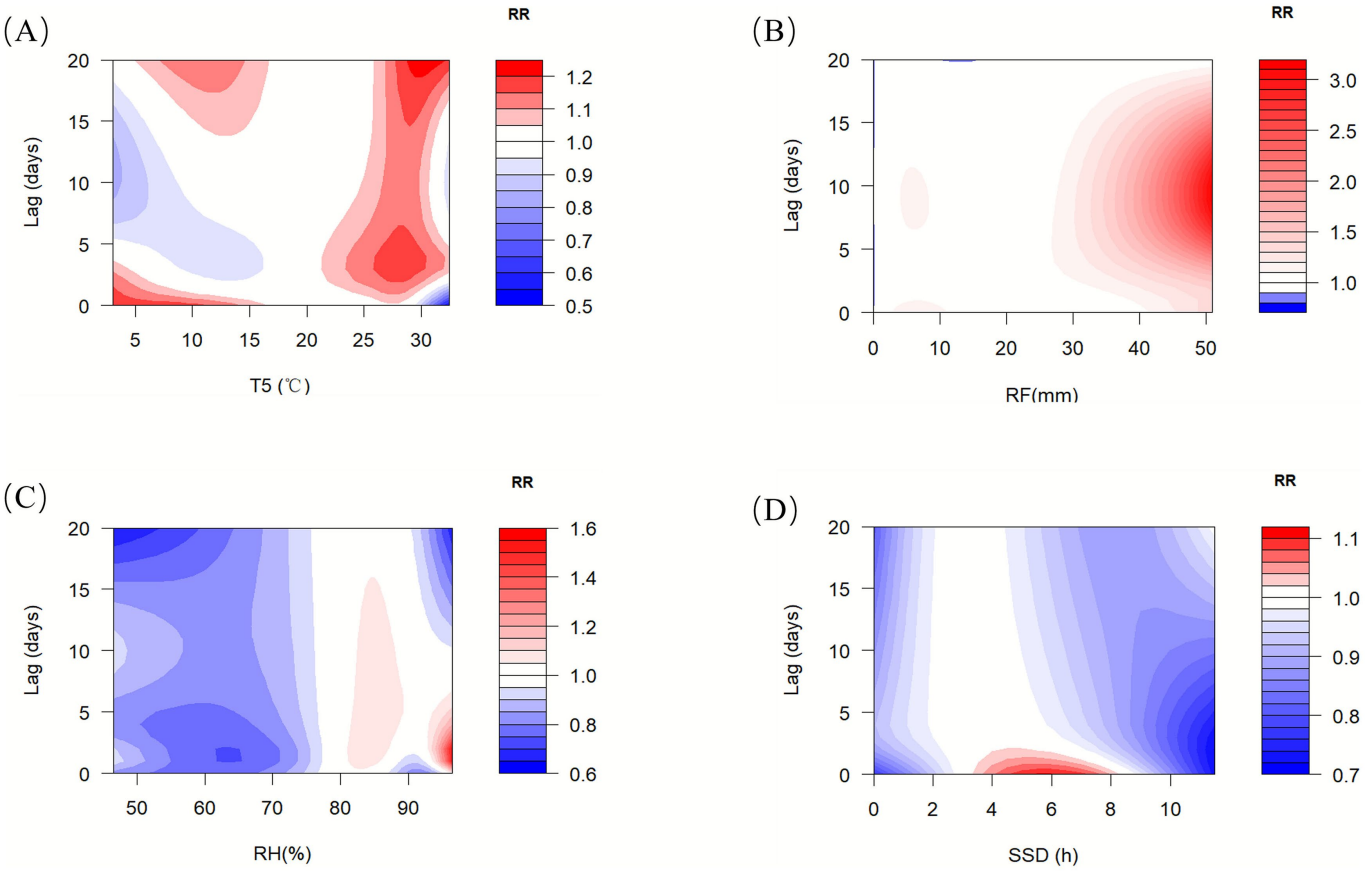
Contour plots for the relative risk of mushroom poisoning along T5 **(A)**, RF **(B)**, RH **(C)**, and SSD **(D)** over different lag days in Guizhou, from 2019 to 2023. RF, rainfall; RH, relative humidity; SSD, sunshine duration; T5, daily average 5 cm ground temperature.

The contour plot in Figure 4 illustrates the associations of various meteorological factors (T5, RF, RH, SSD) and their lag periods on the risk of mushroom poisoning under a single meteorological factor model. When T5 ranged from 25 to 30 °C, it consistently acted as a risk factor for mushroom poisoning throughout the 0–20 day lag period. RF exhibited a harmful association during the lag periods of 5–10 days at 4 mm of rainfall and 0–18 days at >30 mm. Low RH served as a protective factor. In contrast, RH > 80% increased the likelihood of mushroom poisoning but had a weak and short-lived association. SSD was identified as a risk factor only within the 4–8 h range, without any noticeable lag association. In addition, the grouped analysis for rural and urban areas showed a weaker response to changes in meteorological factors in urban areas, with rural areas exhibiting higher sensitivity (Supplementary Figure S6). The results of the multi-factor model analysis were generally consistent with those of the single-factor model (Supplementary Figure S7).

#### 3.4.2 Lagged associations of meteorological factors on the risk of mushroom poisoning at different quartiles

**Figure 5:**
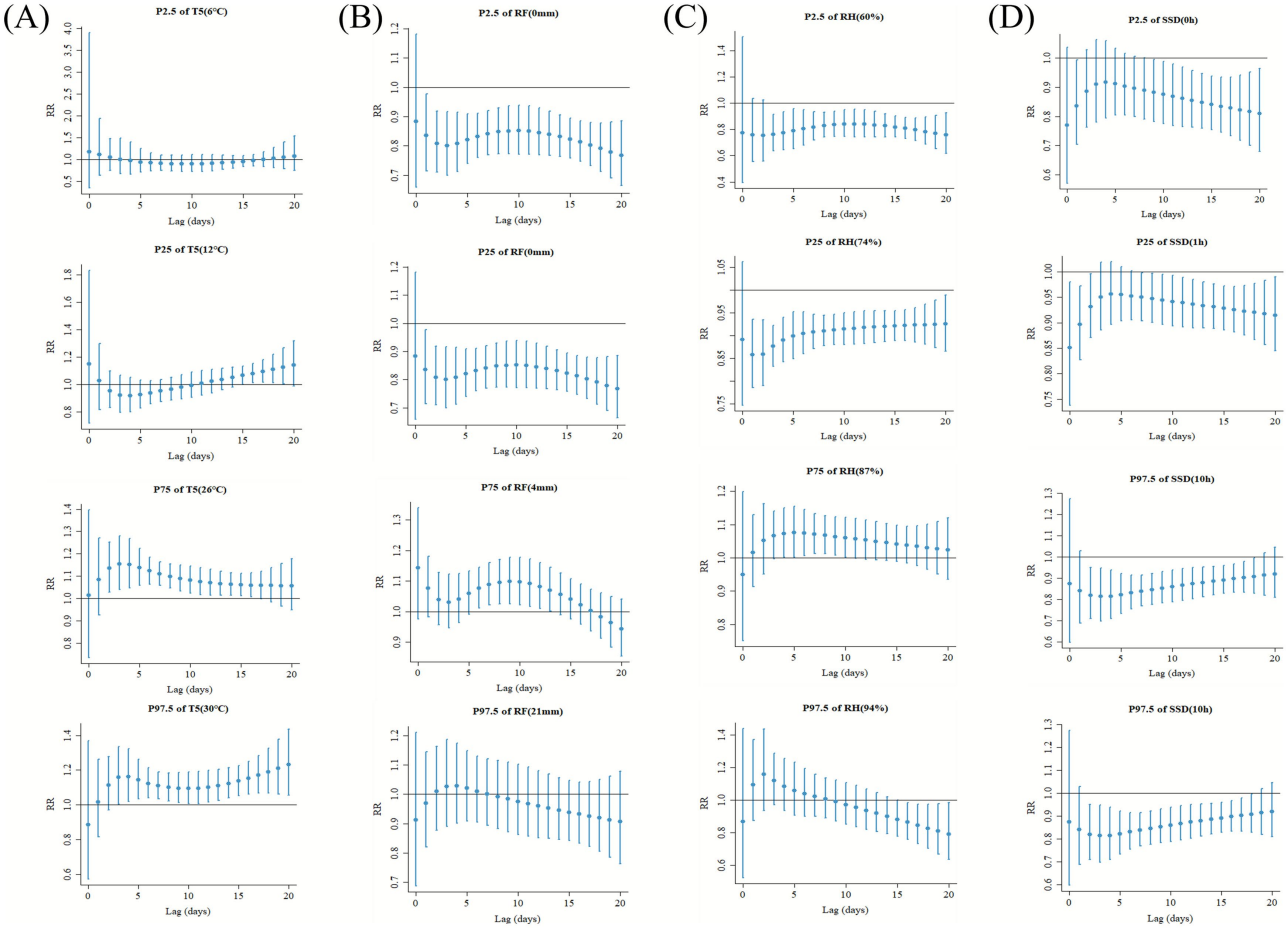
Lagged-specific associations of meteorological factors T5 **(A)**, RF **(B)**, RH **(C)**, and SSD **(D)** on the risk of developing mushroom poisoning at different quartiles. The model was adjusted for PM_2.5_, SO_2_, CO, O_3_. RF, rainfall; RH, relative humidity; SSD, sunshine duration; T5, daily average 5 cm ground temperature.

Figure 5 and Supplementary Figure S8 illustrate the lagged and cumulative associations of T5, RF, RH, and SSD on the risk of mushroom poisoning at different quantiles (P_2.5_, P_25_, P_75_, P_97.5_) in the single meteorological factor model. T5 had no significant association with the risk of mushroom poisoning at the P_2.5_ (6 °C) and P_25_ (12 °C) quantiles. However, at the P_75_ (26 °C) quantile, T5 was a risk factor with a lag of 2 days (RR = 1.13, 95% CI: 1.03–1.25) and continued to be significant up to a lag of 6 days (RR = 1.06, 95% CI: 1.01–1.11). The cumulative risk increased from lag 0–5 (RR = 1.88, 95% CI: 1.10–3.23) to lag 0–20 (RR = 5.53, 95% CI: 2.45–12.47). At the P_97.5_ (30 °C) quantile, the risk further increased, with the highest daily risk at lag 20 (RR = 1.23, 95% CI: 1.06–1.44) and the highest cumulative risk at lag 0–20 (RR = 10.32, 95% CI: 2.89–36.83). RF was a risk factor for mushroom poisoning only at the P_75_ (4 mm) quantile, with the highest single-day risk occurring at lag 9 (RR = 1.10, 95% CI: 1.01–1.18) and the highest cumulative risk at lag 0–17 days (RR = 3.21, 95% CI: 1.31–7.86). RH became a risk factor at the P_75_ (87%) quantile, with the highest daily risk at lag 5 (RR = 1.08, 95% CI: 1.01–1.16) and the highest cumulative risk at lag 0–20 days (RR = 2.56, 95% CI: 1.05–76.23). SSD showed no significant association at the P_75_ (6 h) quantile, but at P_2.5_ (0 h), P_25_ (1 h), and P_97.5_ (10 h) quantiles, it demonstrated a protective association. The results from the multivariate model were generally consistent with those of the univariate model (Supplementary Figure S9).

### 3.5 Associations between interactions of meteorological factors and air pollutants and the risk of mushroom poisoning

**Figure 6:**
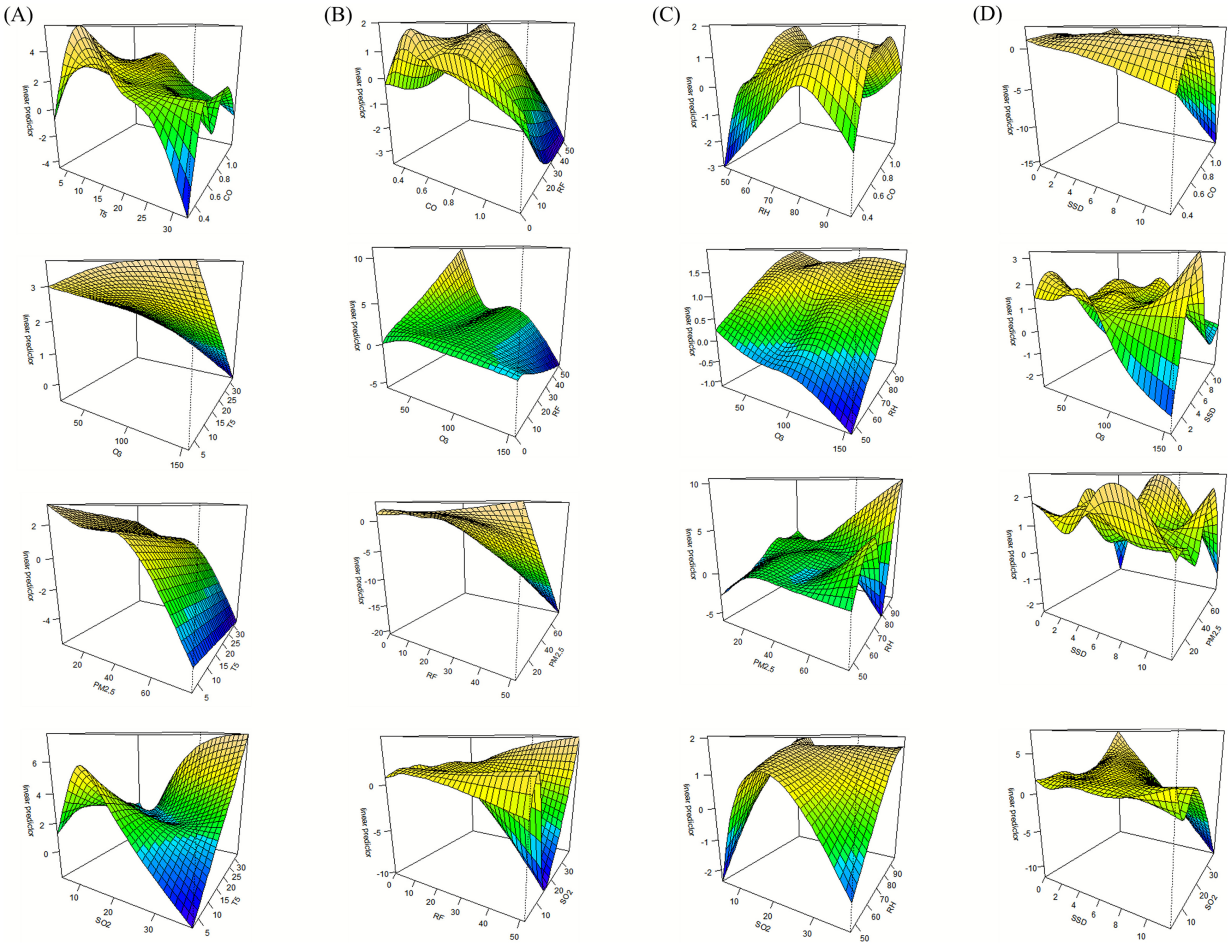
Associations of interactions between meteorological factors and air pollutants on the risk of mushroom poisoning. Interaction of T5 **(A)**, RF **(B)**, RH **(C)**, and SSD **(D)** with CO, O_3_, PM_2.5_, and SO_2_; PM_2.5_, particulate matter 2.5.

Based on the GAM, we evaluated the potential pairwise interactions between meteorological factors (T5, RF, RH, SSD) and air pollutants (CO, PM_2.5_, O_3_, SO_2_) on the risk of mushroom poisoning. The relationships were visualized using three-dimensional diagrams (Figure 6). Figure 6A demonstrates that the risk of mushroom poisoning was significantly elevated under conditions of high T5 combined with high SO_2_ concentrations, as well as low T5 combined with high O_3_ and low PM_2.5_ concentrations, while Figure 6B illustrates that the risk of mushroom poisoning was similarly increased under the combination of high RF with low PM_2.5_ and low O_3_ concentrations. The synergistic association of high RH and high PM_2.5_ concentrations further exacerbated the risk of mushroom poisoning (Figure 6C). Figure 6D indicates that the risk of mushroom poisoning was significantly heightened under conditions of shorter SSD combined with higher SO_2_ concentrations.

### 3.6 Sensitivity analysis

As can be seen from Supplementary Figures S10–S14, varying the degrees of freedom of the exposure-response dimension from 3 to 4 or time from 7 to 8 in the GAM, as well as adding PM_10_ to the multi-air pollutant or multi-meteorological factor DLNM, or changing the maximum lag time to 14 days, did not substantively alter the main results. This suggests that the parameter settings of the GAM and DLNM were reasonably selected and the model fitting results are robust.

## 4 Discussion

Guizhou Province has among the highest incidences of mushroom poisoning in China. From 2019 to 2023, 5,927 mushroom poisoning cases were reported, with rural areas accounting for 72.7% (4,306) and urban areas for 27.3% (1,621). From 2011 to 2023, Guizhou Province reported 97 fatal cases of mushroom poisoning, resulting in a total indirect economic burden of 40.8569 million yuan (approximately 421,200 yuan per case) (13), highlighting the significant threat mushroom poisoning poses to regional public health security. Our previous study employed GAM to analyze 2023 data on meteorological factors and mushroom poisoning cases, revealing significant associations between meteorological conditions and mushroom poisoning incidence, as well as demonstrating that interactions between various meteorological factors could substantially increase poisoning risk (30). Building upon these findings, the present study systematically evaluated the lag associations of daily air pollutants and meteorological factors on mushroom poisoning incidence using time-series data from 2019 to 2023 and investigated the potential interactive associations between air pollutants and meteorological factors on poisoning incidence.

GAM results revealed significant non-linear relationships between air pollutants (CO, O_3_, PM_2.5_, SO_2_), meteorological factors (T5, RF, RH, SSD), and mushroom poisoning incidence. This indicates that the associations of air pollutants and meteorological factors on the risk of mushroom poisoning are not simply monotonic, as traditionally understood, but exhibit complex non-linear characteristics.

Building upon evidence linking environmental factors to disease risks (33–38), we employed a DLNM to quantify the lagged associations of air pollutants and meteorological variables on mushroom poisoning incidence. PM_2.5_, SO_2_, O_3_, and CO concentrations were associated with a decreased risk of mushroom poisoning, with a longer duration in urban areas. We propose that several mechanistic pathways may underlie these associations. From a behavioral exposure pathway perspective, since most poisoning incidents originated from self-picking by the population (14) and considering that existing studies have shown that pollutants such as O_3_, nitrogen oxides, and particulate matter may inhibit the physiological activity and ascospores formation of plants and fungi (39, 40), elevated pollutant concentrations may reduce the risk of mushroom poisoning by inhibiting the growth and reproduction of wild mushrooms, indirectly reducing human exposure. Additionally, rural areas generally have higher vegetation cover, which can absorb pollutants (41). This may partly explain why pollutant impacts in urban areas are more persistent in duration. From a toxicological pathway perspective, studies suggest that pollutants may have associations through direct influence on mushroom toxicity or human susceptibility. For example, *in vitro* toxicological studies have shown that CO exposure may alter the chemical properties of certain mycotoxins (42), whereas human exposure to high concentrations of CO can lead to tissue hypoxia and affect immune function (43, 44). In addition, the strong oxidizing properties of O_3_ interfere with the normal physiological activities of microorganisms (45). This provides indirect support for the hypothesis that it may inhibit mushroom growth. In addition, elevated airborne concentrations of PM_2.5_ and SO_2_ increase damage to humans and other organisms, and contribute significantly to disease burden (46–49), further confirming that elevated concentrations of airborne pollutants result in a reduced risk of mushroom poisoning. It should be noted that the above explanation of the mechanism of “protective association” of pollutants by inhibiting the growth of mushrooms or altering their toxicity remains hypothetical and needs to be verified by standardized experimental studies.

Regarding meteorological factors, the results showed that T5 did not have a significant association with the risk of mushroom poisoning at the P_2.5_ (6 °C) and P_25_ (12 °C) quartiles, but was a risk factor at the P_75_ (26 °C) and P_97.5_ (30 °C) quartiles, explaining the high prevalence of mushroom poisoning in the summer and autumn, as observed in previous studies (14, 30, 50). Moreover, the present study demonstrated that the association of higher temperature with the risk of mushroom poisoning had a lag and cumulative lag association, and persisted for a long duration. In addition, both RF and RH showed protective associations at the P_2.5_ and P_25_ quartiles, with dangerous associations at the P_75_ quartile. While previous studies showed that rainfall and relative humidity are risk factors for mushroom poisoning (28, 30, 51), the present study refined the associations of rainfall and relative humidity on mushroom poisoning and considered the hysteresis association, revealing their lagged and cumulative lag associations. Specifically, rainfall and relative humidity only become risk factors for mushroom poisoning when they are at moderate levels, providing a basis for the accurate prevention and control of mushroom poisoning. It is worth noting that our study found that too high or too low SSD can reduce the occurrence of mushroom poisoning, indicating that the growth of mushrooms depends on the SSD, with too high or too low SSD not conducive to mushroom growth. When SSD was within the range of 4–8 h, the risk of mushroom poisoning increased, and this effect did not have a hysteresis association, in line with the existing understanding (14, 30). This reflects the specific dependence of mushroom growth on light conditions.

Meteorological factors had a significantly stronger influence on the occurrence of mushroom poisoning in rural areas than in urban areas. We believe that this urban–rural difference is the result of a combination of behavioral patterns, environmental exposures, and demographic characteristics. At the behavioral level, residents in rural areas rely more on wild harvesting as a source of mushrooms (9, 18), and their exposure opportunities are directly regulated by meteorological conditions, whereas urban residents mainly purchase from the market and are less affected by real-time meteorological fluctuations. At the level of environmental exposure, higher vegetation cover and more complex local microclimates (e.g., understory humidity, temperature gradients) in rural areas (41) may enhance the direct modulation of mushroom growth by meteorological factors. In addition, demographic differences should not be ignored: a higher proportion of young adults in rural areas being engaged in agriculture and outdoor work means more opportunities to participate in foraging activities (52); at the same time, the traditional foraging practices retained by some rural elders may maintain the continuation of such behaviors. In addition, the unequal distribution of resources for public health promotion between urban and rural areas may affect the effectiveness of risk communication and responses (53, 54). In conclusion, urban–rural differences are not caused by a single factor but are shaped by a combination of behavioral-environmental-demographic factors, which is important for the development of differentiated prevention and control strategies.

Using GAM, this study investigated the combined associations of meteorological factors (T5, RF, RH, and SSD) and air pollutants (CO, O_3_, PM_2.5_, SO_2_) on mushroom poisoning risk. Our analysis revealed that specific interactions between these environmental factors significantly elevate poisoning incidence. Particularly noteworthy were the synergistic associations observed between high T5 and elevated SO_2_ levels, as well as between low T5 conditions and the combination of high O_3_ with low PM_2.5_ concentrations. Similarly, periods of heavy RF coupled with reduced PM_2.5_ and O_3_ levels demonstrated increased risk, while high RH environments with elevated PM_2.5_ concentrations and low SSD conditions with high SO_2_ exposure also showed significant associations. Although air pollutant concentrations alone exhibited no independent correlation with poisoning risk, the findings demonstrate substantial modifying associations when interacting with meteorological conditions. While such interaction mechanisms have not been previously documented for mushroom poisoning, their existence is well-established in analogous environmental health contexts (55–57).

This study systematically revealed the independent, lagged, and interactive associations of air pollutants and meteorological factors on the risk of mushroom poisoning in Guizhou Province, offering new insights into environmentally driven mechanisms. However, the conclusions need to be interpreted considering the unique cultural context of Guizhou. The local tradition of wild mushroom foraging in the local multi-ethnic population significantly enhances the reality of environmental factors on the risk of poisoning, which may not apply to regions lacking a similar dietary culture.

Several limitations should also be acknowledged. (1) the study did not control for potential confounders such as foraging behavior and socio-economic changes; (2) the reliance on passive surveillance data, possibly leading to reporting bias; and (3) the ecological design limits the ability to fully capture spatial heterogeneity. Despite these limitations, the study provides valuable public health insights. For the first time, the independent, lagged, and interactive associations of environmental factors on mushroom poisoning were systematically quantified and the behavioral mechanisms behind the urban–rural differences were revealed. The results can be used to optimize early warning systems in high-incidence areas such as Guizhou, to achieve accurate risk prediction and differential prevention and control through the integration of key meteorological thresholds and pollutant data. This will allow public health resources to be allocated more efficiently to reduce the disease burden of mushroom poisoning.

## 5 Conclusion

Our study uncovered a complex interplay between meteorological factors, air pollutants, and the risk of mushroom poisoning in Guizhou Province, China. The results demonstrated that meteorological factors (T5, RF, RH, and SSD) and air pollutants (CO, PM_2.5_, O_3_, and SO_2_) exhibited significant nonlinear relationships with the number of mushroom poisoning cases. Furthermore, these factors influenced mushroom poisoning through both lagged and cumulative associations, with varying temporal durations. Notably, interactions between meteorological factors and air pollutants further amplified the risk of mushroom poisoning. These findings provide scientific evidence for the precise prevention and control of mushroom poisoning.

## Data Availability

The data analyzed in this study is subject to the following licenses/restrictions: Data involves patient privacy and is not publicly available. Requests to access these datasets should be directed to guohua_cqy@163.com.

## Footnotes

1
https://www.tianditu.gov.cn/

2
https://air.cnemc.cn:18007

3
http://gz.cma.gov.cn/
