# Supplementary material for: The association between air pollutants, meteorological factors, and mushroom poisoning cases in Guizhou Province, China: a 5-year time-series study
Source: Front Public Health. 2026 Jan 8;13:1699557. doi: 10.3389/fpubh.2025.1699557 (PMC12823814; doi:10.3389/fpubh.2025.1699557)
Supplement: Supplementary file 1 [file Data_Sheet_1.zip › Supplementary Material(最终).docx]

**
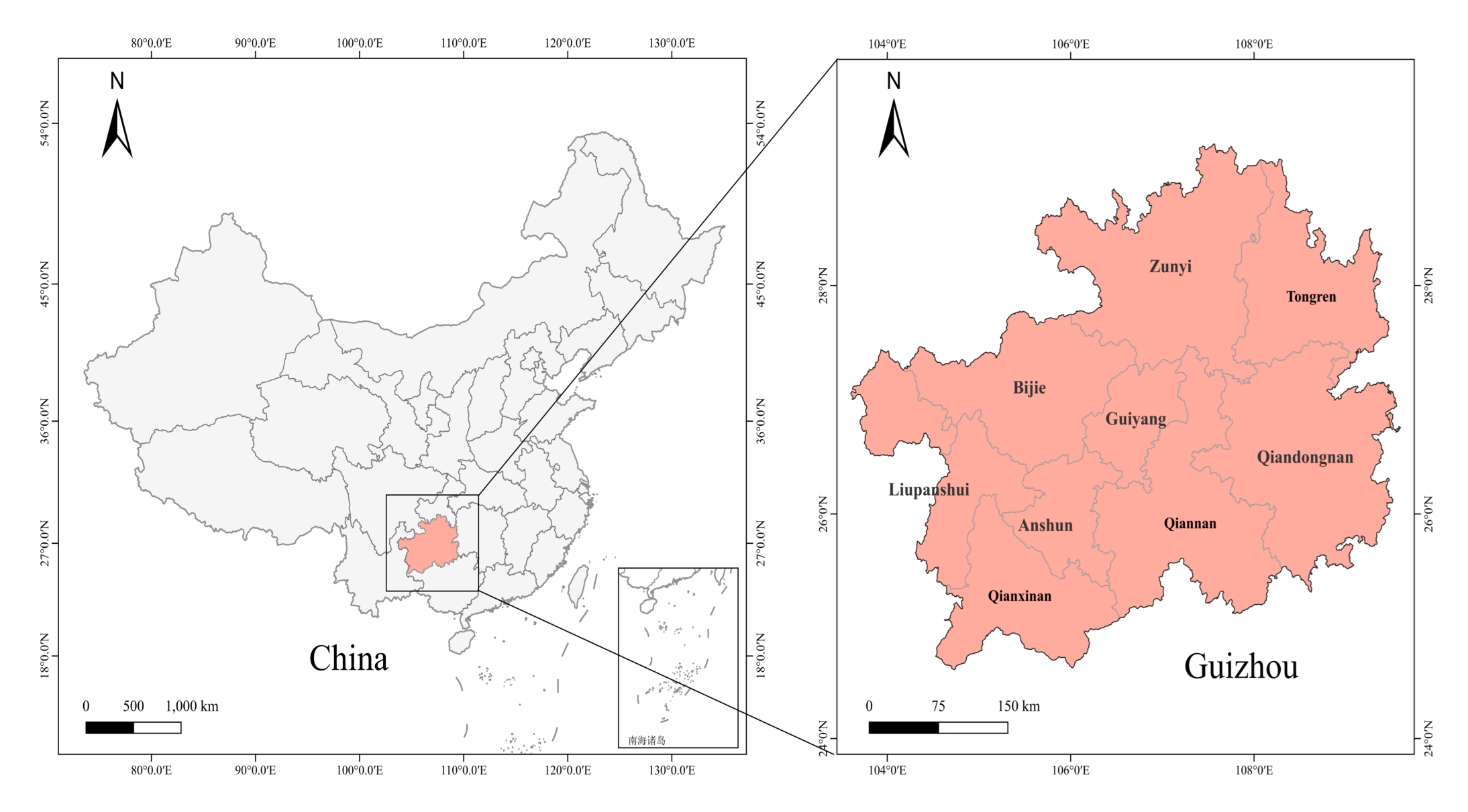
Fig. S1** Location of Guizhou Province within the geographical region of China.


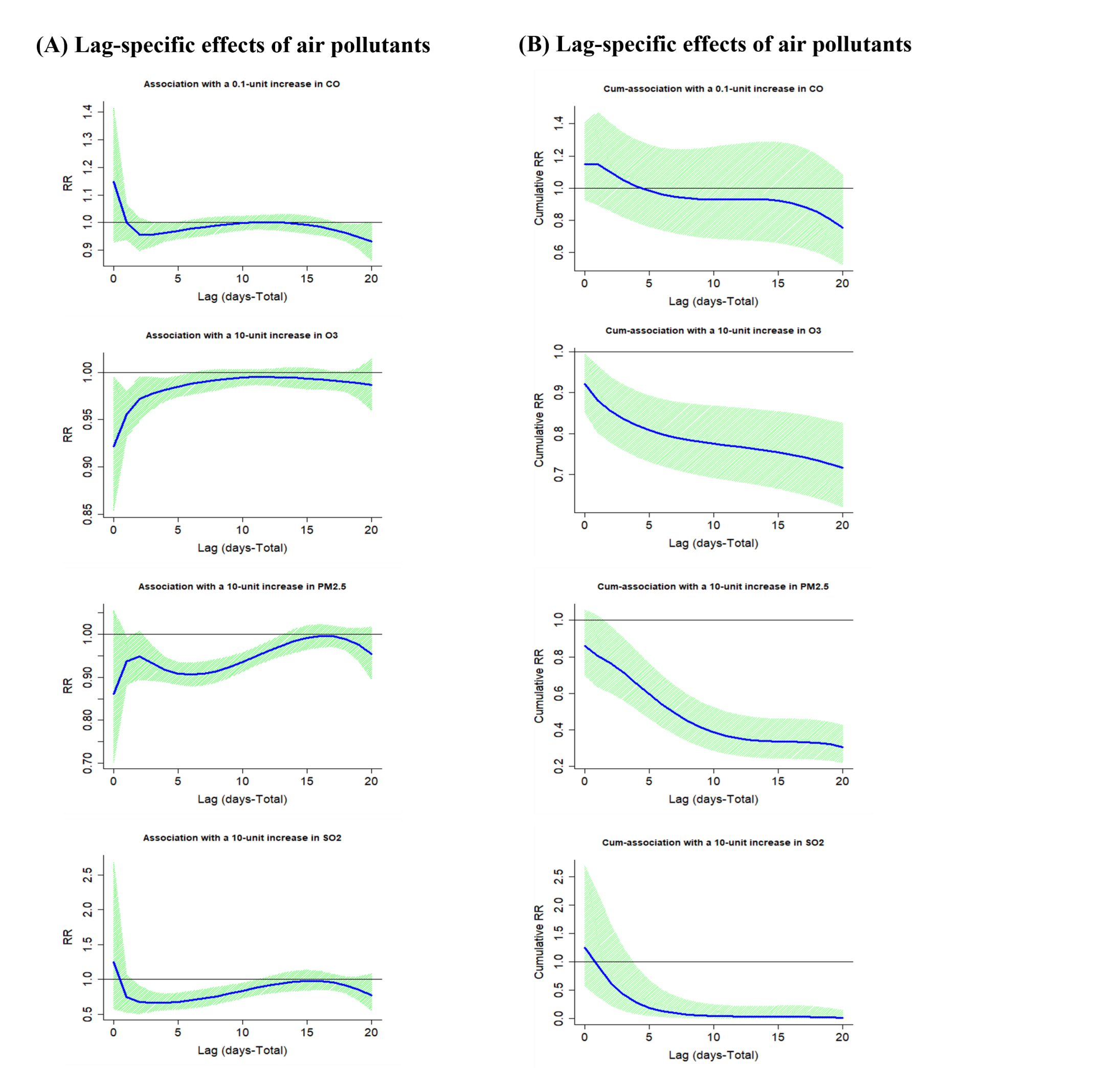


**Fig. S2** Effect of air pollutants (CO, O_3_, PM_2.5_, SO_2_) on the risk of mushroom poisoning at specific**(A)** and cumulative**(B)** lag times. Model was constructed by PM_2.5_, SO_2_, CO, O_3_, T5, RF, RH, and SSD. Abbreviations: RF, rainfall; RH, relative humidity; SSD, sunshine duration; T5, daily average 5 cm ground temperature; PM2.5, Particulate Matter 2.5.


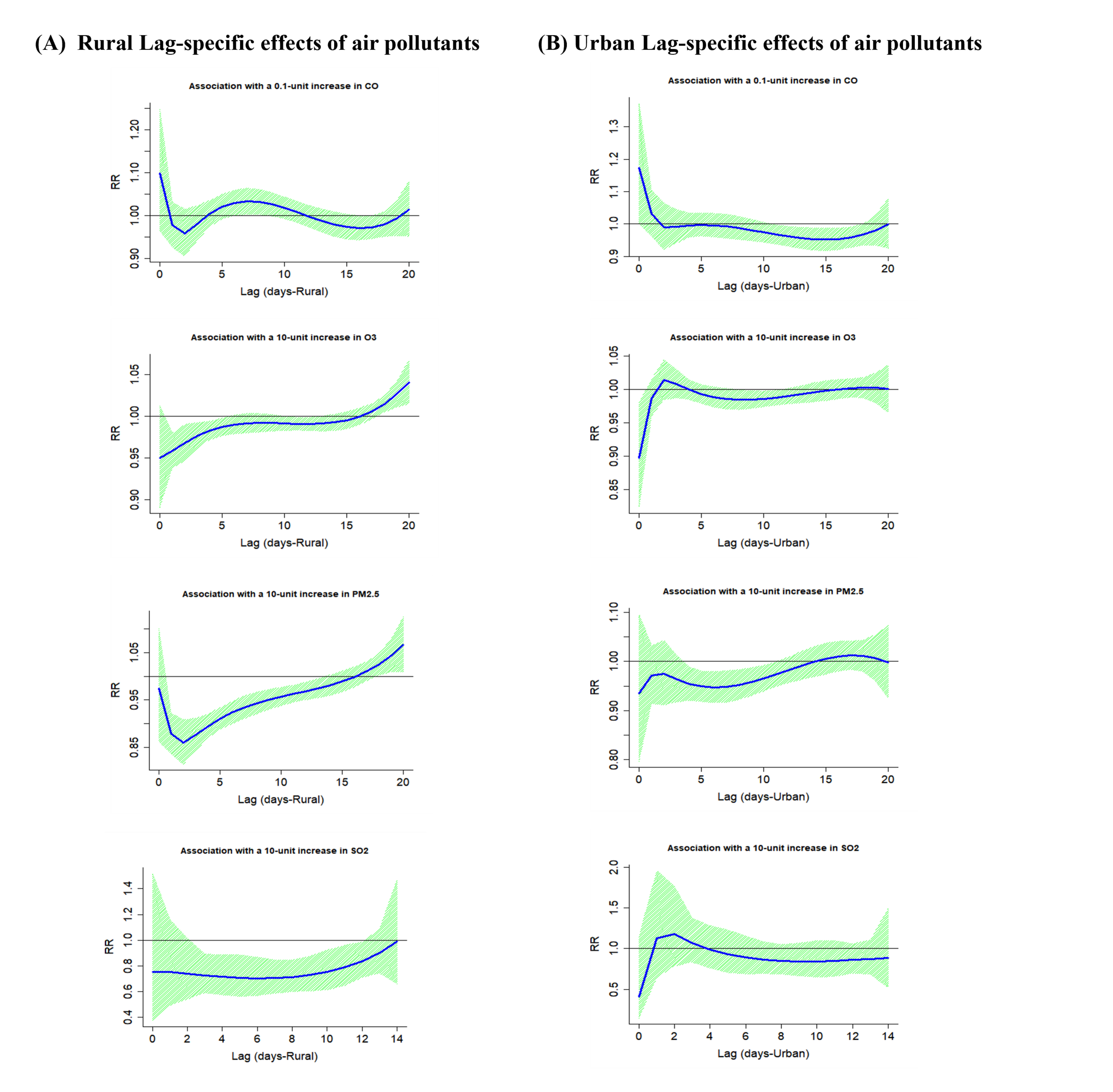


**Fig. S3** Effect of air pollutants (CO, O_3_, PM_2.5_, SO_2_) on the risk of mushroom poisoning in rural **(A)** and urban **(B)** areas at specific lag times. Model was adjusted for T5, RF, RH, and SSD. Abbreviations: RF, rainfall; RH, relative humidity; SSD, sunshine duration; T5, daily average 5 cm ground temperature; PM2.5, Particulate Matter 2.5.


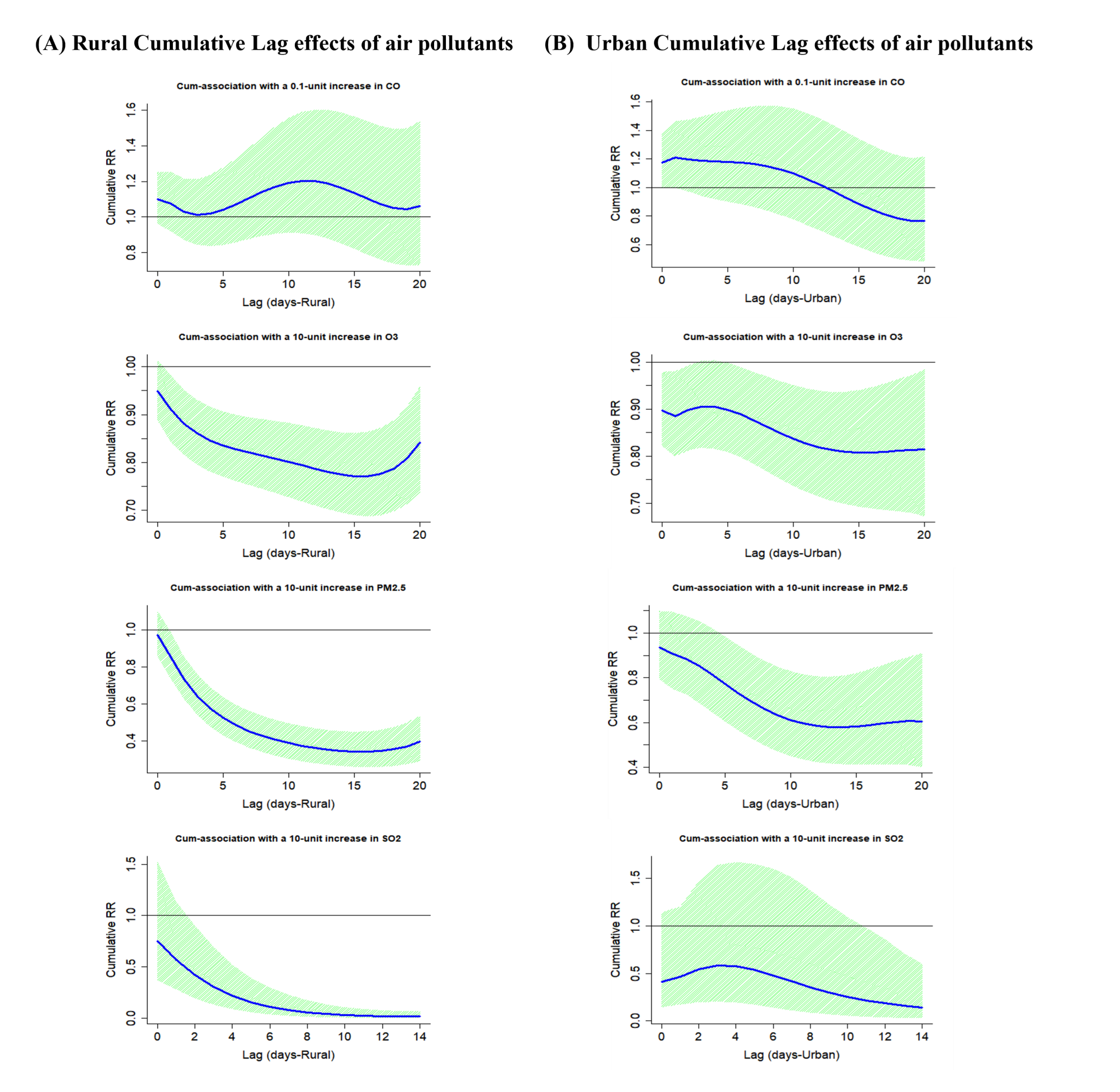


**Fig. S****4** Effect of air pollutants (CO, O_3_, PM_2.5_, SO_2_) on the risk of mushroom poisoning in rural (A) and urban (B) areas at cumulative lag times. Model was adjusted for T5, RF, RH, and SSD. Abbreviations: RF, rainfall; RH, relative humidity; SSD, sunshine duration; T5, daily average 5 cm ground temperature; PM2.5, Particulate Matter 2.5.


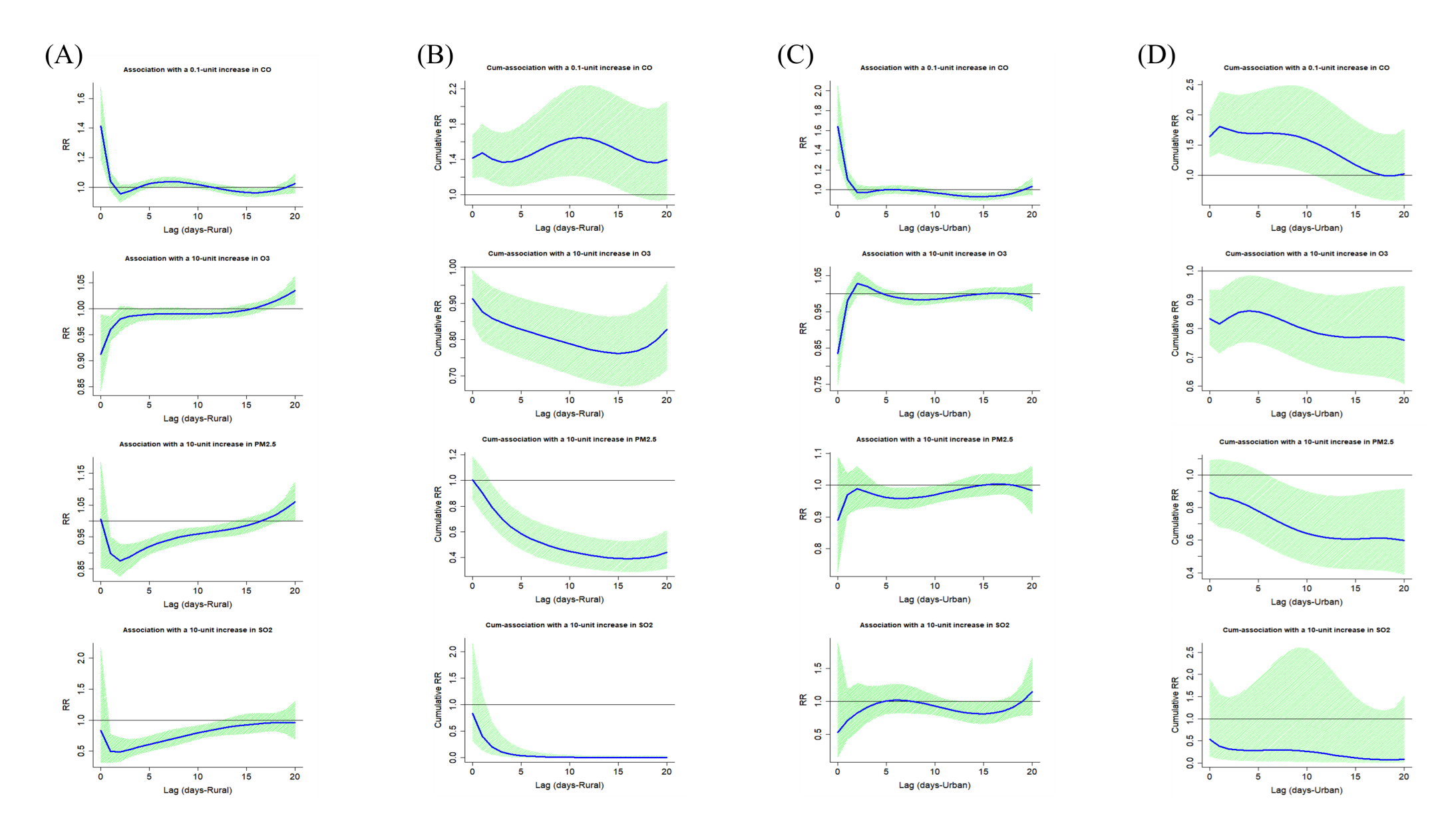


**Fig. S5** Effect of air pollutants (CO, O_3_, PM_2.5_, SO_2_) on the risk of mushroom poisoning in rural (**A, B**) and urban (**C, D**) areas at single-day and cumulative lag times. Model was constructed by PM_2.5_, SO_2_, CO, O_3_, T5, RF, RH, and SSD. Abbreviations: RF, rainfall; RH, relative humidity; SSD, sunshine duration; T5, daily average 5 cm ground temperature; PM2.5, Particulate Matter 2.5.


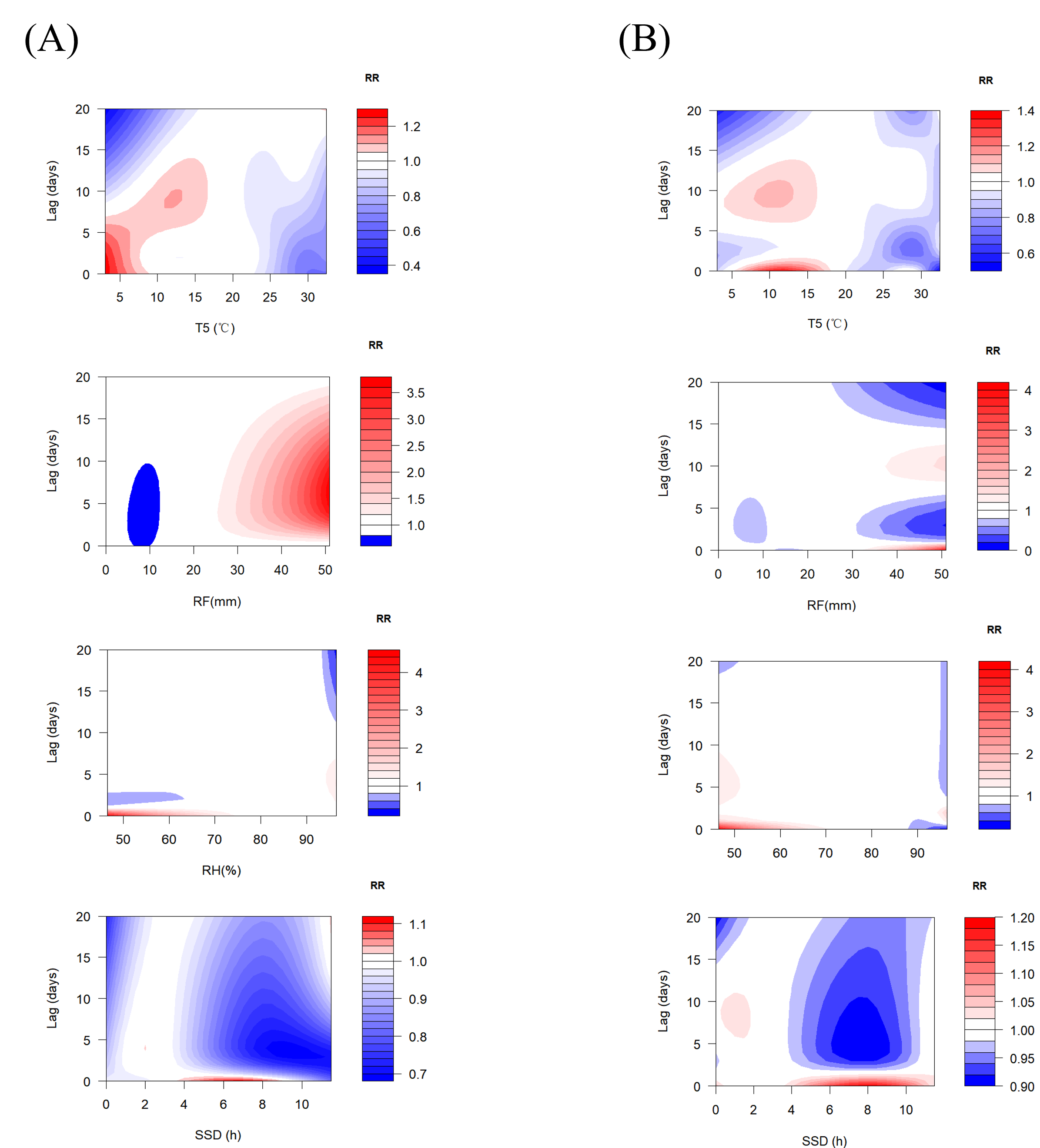


**Fig. S****6** Lagged effects of meteorological factors (T5, RF, RH, SSD) on the risk of mushroom poisoning occurrence in rural **(****A)** and urban **(B)** areas. Model was adjusted for PM_2.5_, SO_2_, CO, O_3_. Abbreviations: RF, rainfall; RH, relative humidity; SSD, sunshine duration; T5, daily average 5 cm ground temperature; PM2.5, Particulate Matter 2.5.


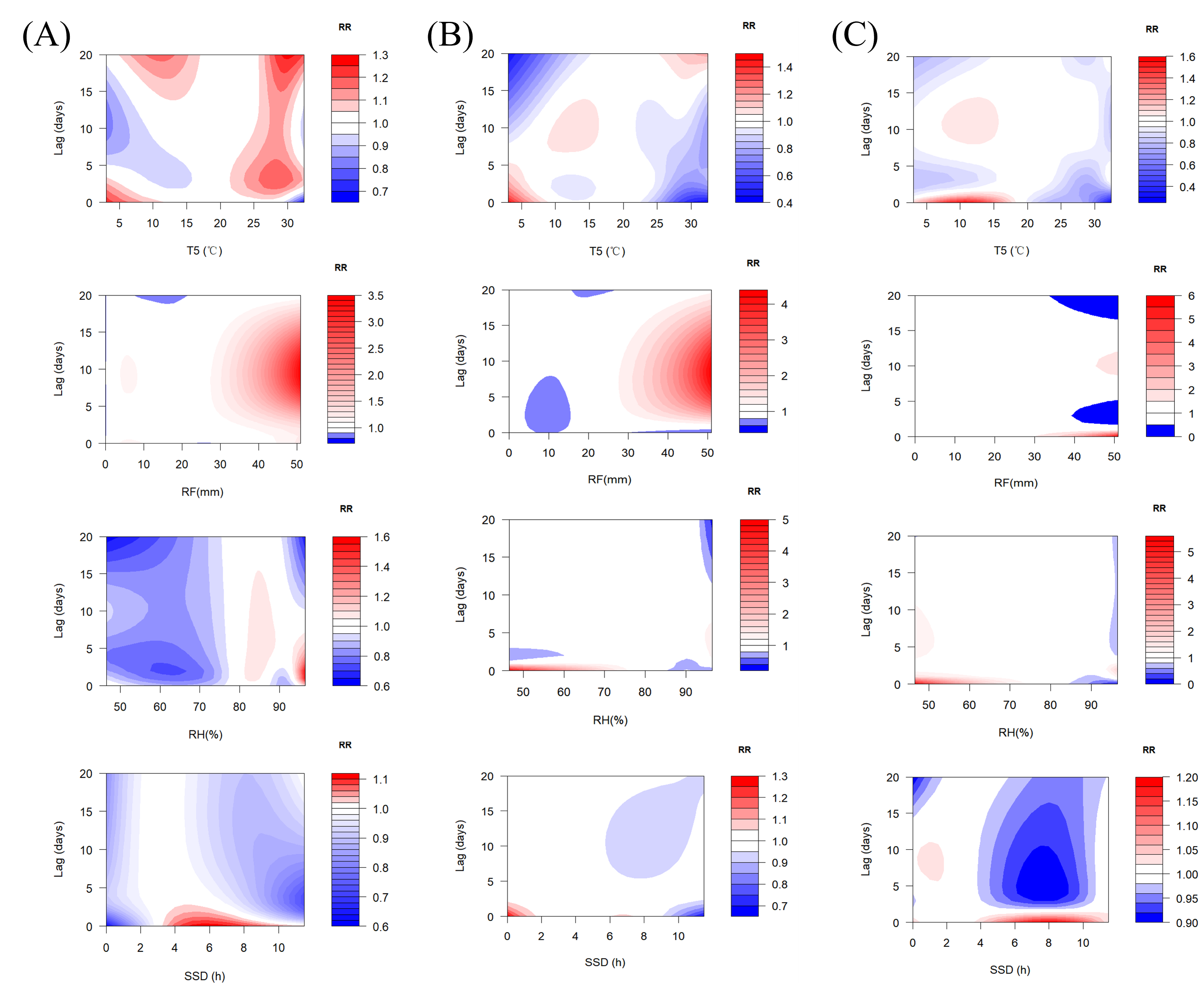


**Fig. S7** Lagged effects of meteorological factors (T5, RF, RH, SSD) on the risk of mushroom poisoning occurrence in the total population **(A)**, rural **(B)**, and urban **(C)** areas. Model was constructed by PM_2.5_, SO_2_, CO, O_3_, T5, RF, RH, and SSD. Abbreviations: RF, rainfall; RH, relative humidity; SSD, sunshine duration; T5, daily average 5 cm ground temperature; PM2.5, Particulate Matter 2.5.


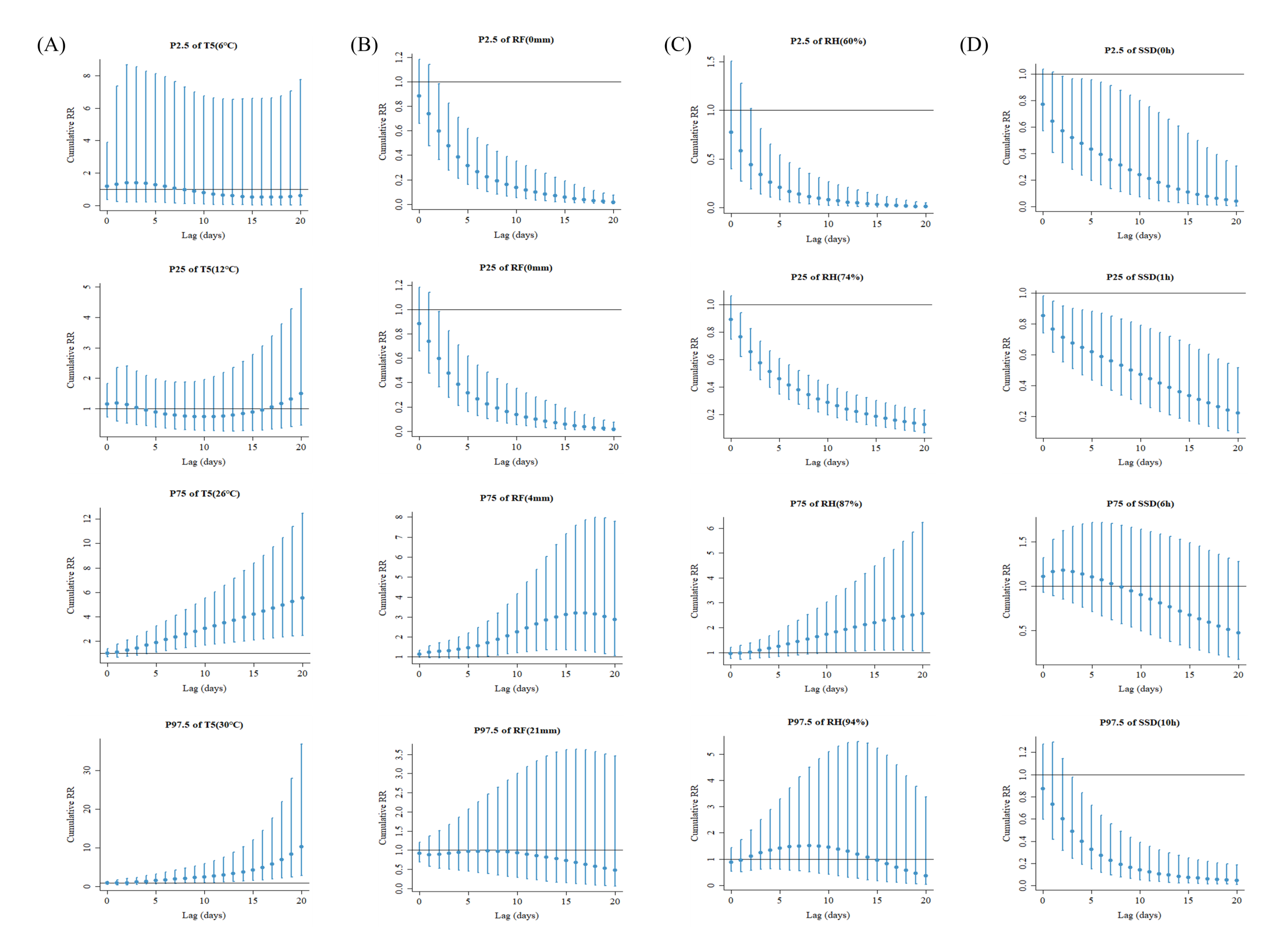


**Fig. S8** Lagged-cumulative effects of meteorological factors T5 **(A)**, RF **(B)**, RH **(C)** and SSD **(D)** on the risk of developing mushroom poisoning at different quartiles. Model was adjusted for PM_2.5_, SO_2_, CO, O_3_. Abbreviations: RF, rainfall; RH, relative humidity; SSD, sunshine duration; T5, daily average 5 cm ground temperature; PM2.5, Particulate Matter 2.5.


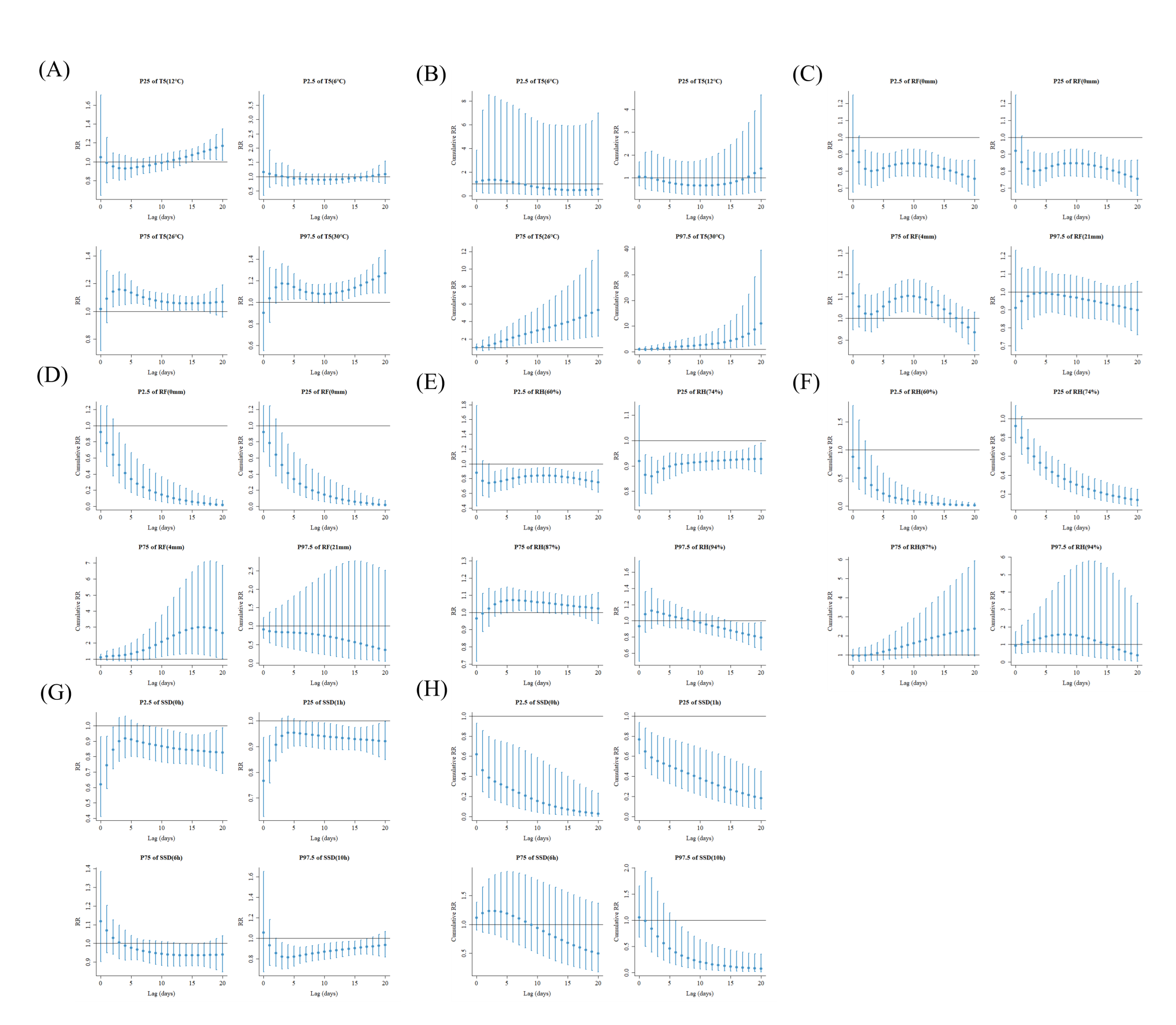


**Fig. S9** Lagged effects of meteorological factors T5 (A, B), RF (C, D), RH (E, F) and SSD (G, H) on the risk of developing mushroom poisoning at different quartiles. Model was constructed by PM_2.5_, SO_2_, CO, O_3_, T5, RF, RH, and SSD. Abbreviations: RF, rainfall; RH, relative humidity; SSD, sunshine duration; T5, daily average 5 cm ground temperature; PM2.5, Particulate Matter 2.5.


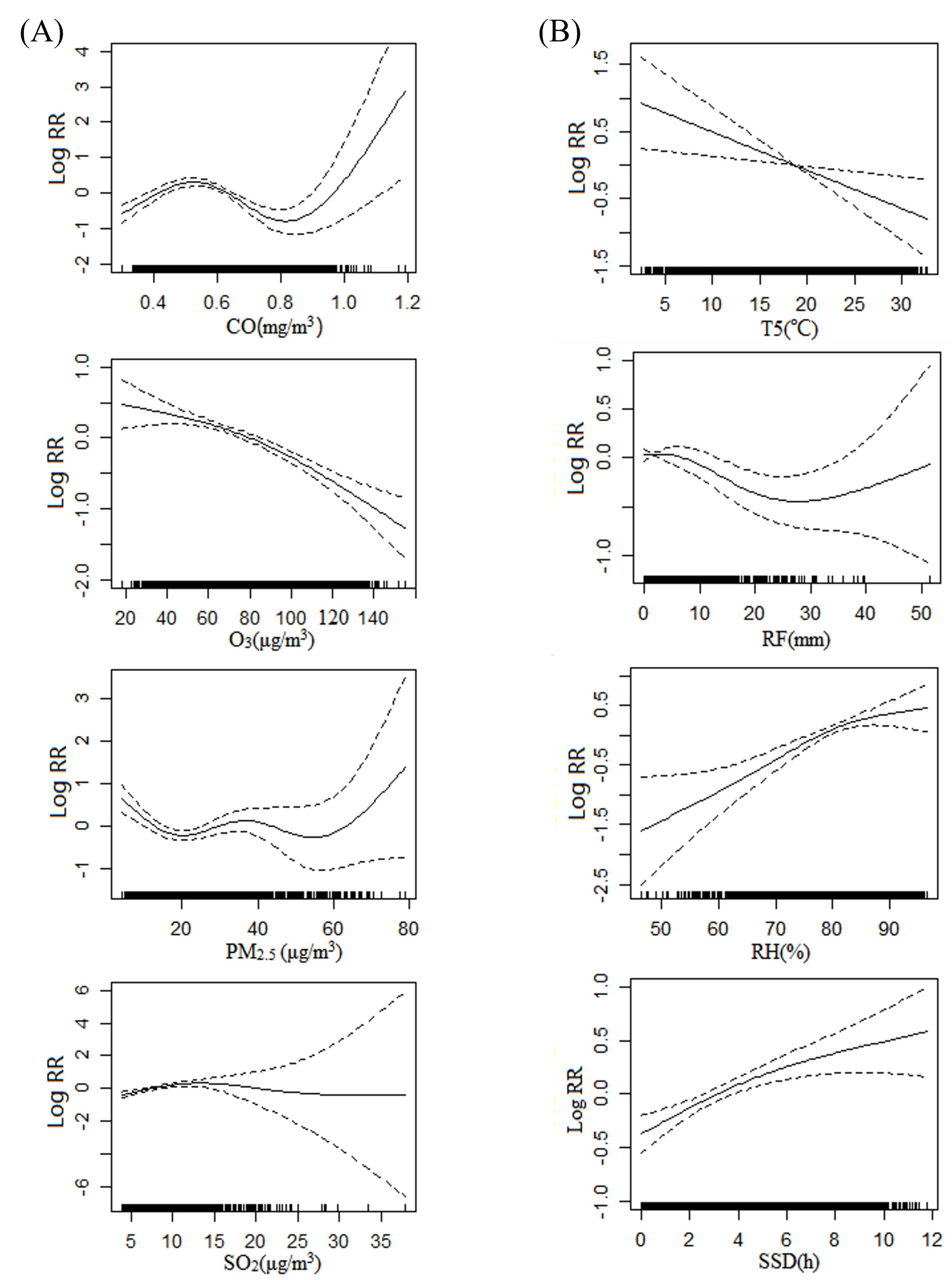


**Fig. S10** Exposure-response relationship between meteorological factors (**A:** T5, RF, RH, SSD), air pollutants (**B:** CO, O_3_, PM_2.5_, SO_2_) and the number of mushroom poisoning cases when changing the parameter settings of the exposure dimension degrees of freedom in the GAM model (3 to 4). The solid lines indicate the logarithmic relative risk of mushroom poisoning and the dotted lines indicate the 95% confidence level. Abbreviations: RF, rainfall; RH, relative humidity; SSD, sunshine duration; T5, daily average 5 cm ground temperature; PM2.5, Particulate Matter 2.5.


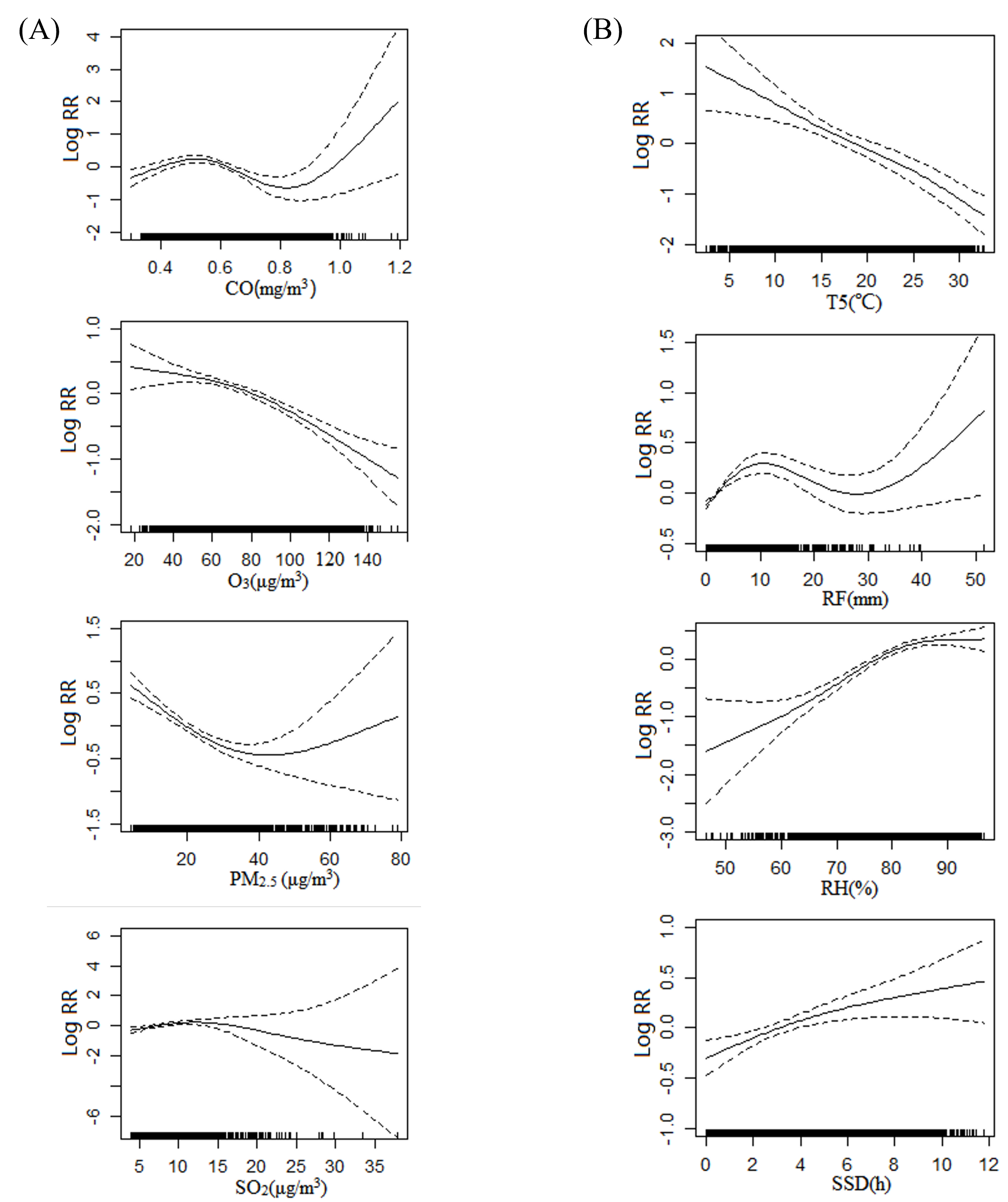


**Fig. S11** Exposure-response relationships between meteorological factors (**A:** T5, RF, RH, SSD), air pollutants (**B:** CO, O_3_, PM_2.5_, SO_2_) and the number of mushroom poisoning cases when changing the parameter settings of time degrees of freedom in the GAM model (7 to 8). Abbreviations: RF, rainfall; RH, relative humidity; SSD, sunshine duration; T5, daily average 5 cm ground temperature; PM2.5, Particulate Matter 2.5.


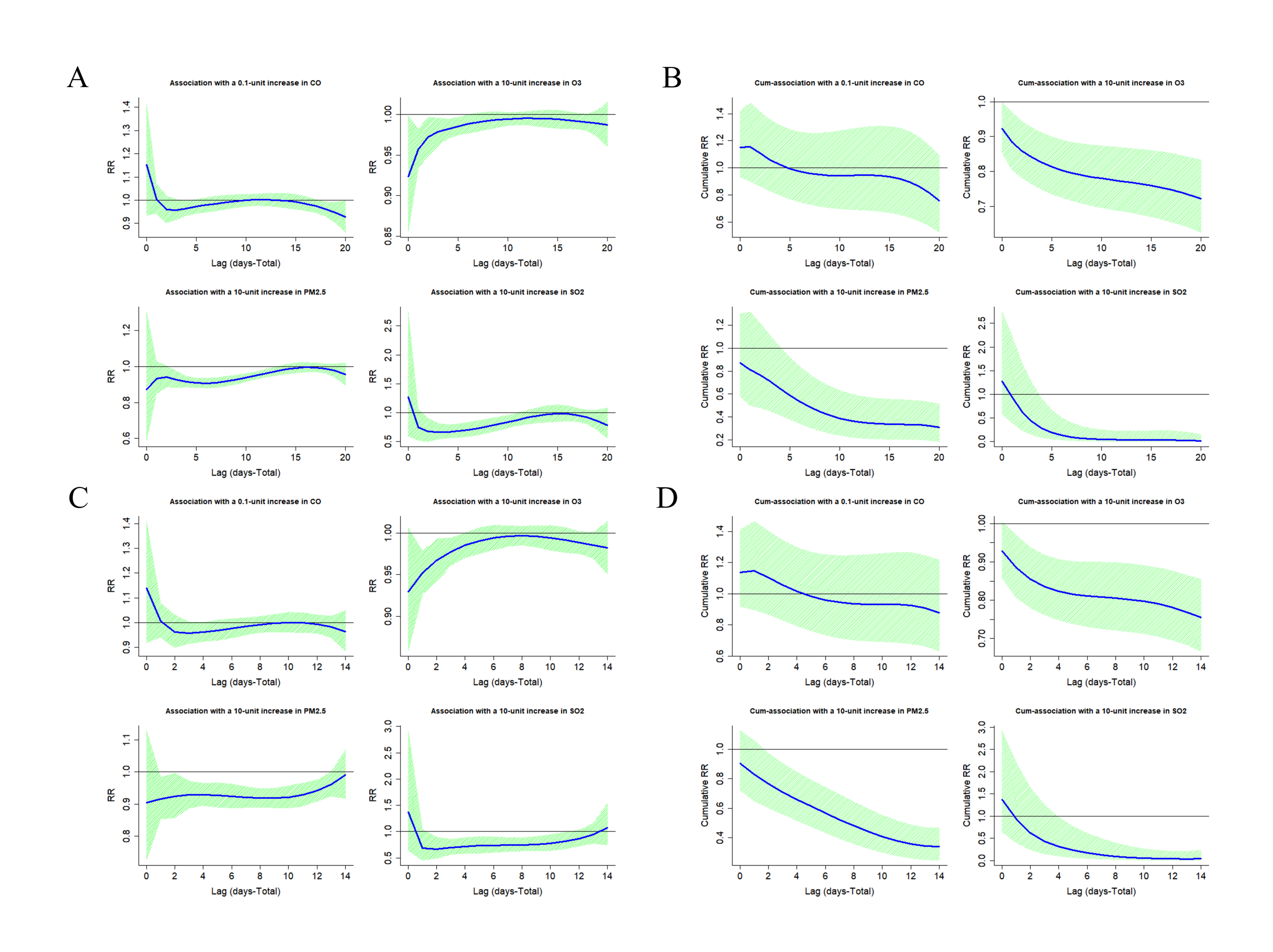


**Fig. S12** Effect of adding the variable PM_10_ in the multifactorial DLNM model (**A, B**) and varying the maximum number of lag days to 14 days (**C, D**) on the risk of mushroom poisoning by air pollutants (CO, O_3_, PM_2.5_, SO_2_) in single-day and cumulative lag time. Abbreviations: RF, rainfall; RH, relative humidity; SSD, sunshine duration; T5, daily average 5 cm ground temperature; PM2.5, Particulate Matter 2.5.


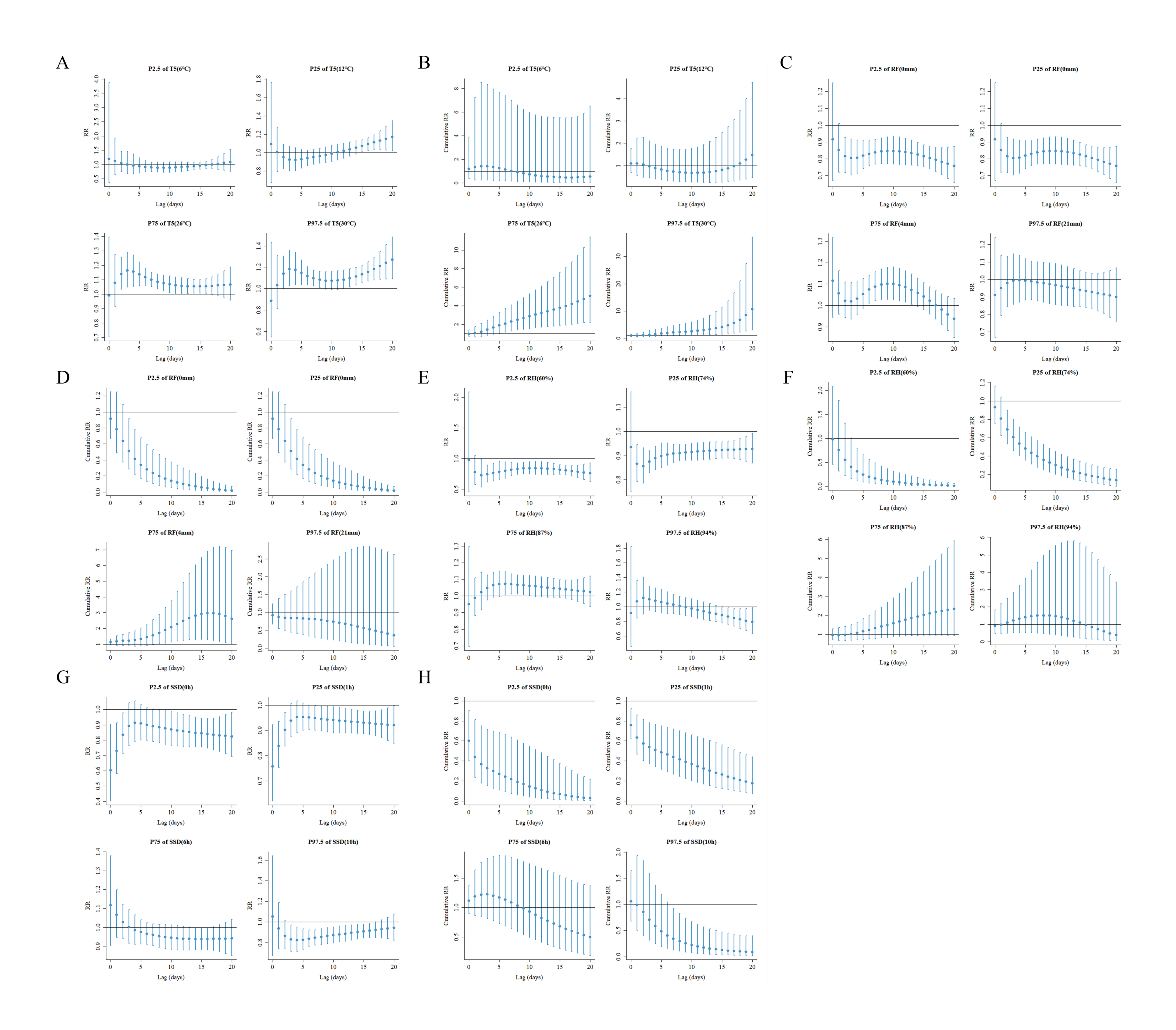


**Fig. S13** Lagged effects of meteorological factors on the risk of mushroom poisoning at different quartiles when the variable PM_10_ was added to the multifactorial DLNM model. Model was constructed by PM_10_, PM_2.5_, SO_2_, CO, O_3_, T5, RF, RH, and SSD. Abbreviations: RF, rainfall; RH, relative humidity; SSD, sunshine duration; T5, daily average 5 cm ground temperature; PM2.5, Particulate Matter 2.5.

**
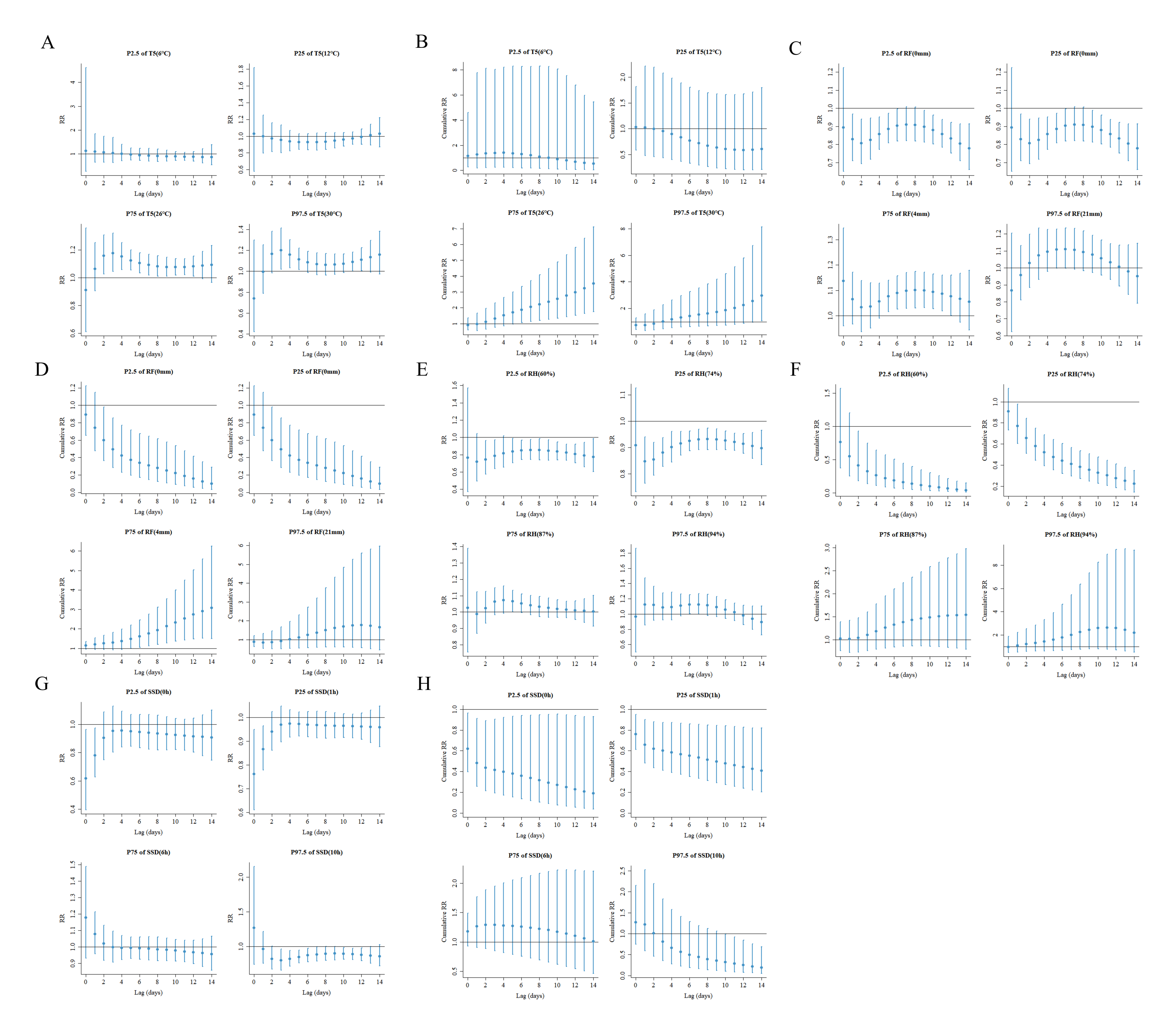
**

**Fig. S14** Lagged effect of meteorological factors on the risk of mushroom poisoning when the maximum number of lag days is 14 in a multifactorial DLNM model. Model was constructed by PM_2.5_, SO_2_, CO, O_3_, T5, RF, RH, and SSD. Abbreviations: RF, rainfall; RH, relative humidity; SSD, sunshine duration; T5, daily average 5 cm ground temperature; PM2.5, Particulate Matter 2.5.

**Table S1** Spearman correlation analysis between air pollutants and meteorological factors

| Variable | AT | RF | Rh | Sun | AW | T5 | PM_2.5_ | PM_10_ | SO_2_ | NO_2_ | O_3_ | CO |
| --- | --- | --- | --- | --- | --- | --- | --- | --- | --- | --- | --- | --- |
| AT | 1.00 |  |  |  |  |  |  |  |  |  |  |  |
| RF | 0.20^*^ | 1.00 |  |  |  |  |  |  |  |  |  |  |
| Rh | -0.26^*^ | 0.62^*^ | 1.00 |  |  |  |  |  |  |  |  |  |
| SSD | 0.59^*^ | -0.39^*^ | -0.74^*^ | 1.00 |  |  |  |  |  |  |  |  |
| AW | 0.15^*^ | 0.03 | -0.17^*^ | 0.05^*^ | 1.00 |  |  |  |  |  |  |  |
| T5 | 0.99^*^ | 0.21* | -0.26^*^ | 0.59^*^ | 0.12^*^ | 1.00 |  |  |  |  |  |  |
| PM_2.5_ | -0.52^*^ | -0.51^*^ | -0.30^*^ | 0.00 | -0.20^*^ | -0.53^*^ | 1.00 |  |  |  |  |  |
| PM_10_ | -0.53^*^ | -0.53^*^ | -0.41^*^ | 0.11^*^ | -0.21^*^ | -0.41^*^ | 0.97^*^ | 1.00 |  |  |  |  |
| SO_2_ | -0.61^*^ | -0.34^*^ | -0.04 | -0.18^*^ | -0.32^*^ | -0.61^*^ | 0.64^*^ | 0.52^*^ | 1.00 |  |  |  |
| NO_2_ | -0.41^*^ | -0.30^*^ | -0.10^*^ | -0.03 | -0.53^*^ | -0.41^*^ | 0.63^*^ | 0.68^*^ | 0.73^*^ | 1.00 |  |  |
| O_3_ | 0.47^*^ | -0.20^*^ | -0.65^*^ | 0.68^*^ | 0.07^*^ | 0.48^*^ | 0.17^*^ | 0.28^*^ | -0.16^*^ | 0.03 | 1.00 |  |
| CO | -0.68^*^ | -0.20^*^ | 0.24^*^ | -0.35^*^ | -0.28^*^ | -0.70^*^ | 0.64^*^ | 0.55^*^ | 0.67^*^ | 0.59^*^ | -0.27^*^ | 1.00 |

^*^*P<*0.05. Abbreviations: AT, average daily temperature; AW, average wind speed; RF, rainfall; RH, relative humidity; SSD, sunshine duration; T5, daily average 5 cm ground temperature; PM_2.5,_ Particulate Matter 2.5_;_ PM_10,_ Particulate Matter10_._

**Table S2** The association between an increase of every 0.1 mg/m³ of CO and mushroom

poisoning in Guizhou Province from 2019 to 2023.

| **Single-day** | **Relative risk**  **(95%, confidence interval)** | **Multi-day** | **Relative risk**  **(95%, confidence interval)** |
| --- | --- | --- | --- |
| 0 | 0.98 (0.83-1.16) | 0-0 | 0.98(0.83-1.16) |
| 1 | 0.97 (0.91-1.03) | 0-1 | 0.94 (0.77-1.16) |
| 2 | 0.96 (0.90-1.03) | 0-2 | 0.91(0.74-1.12) |
| 3 | 0.97 (0.92-1.02) | 0-3 | 0.88 (0.71-1.09) |
| 4 | 0.97 (0.93-1.01) | 0-4 | 0.85 (0.68-1.07) |
| 5 | 0.97 (0.94-1.04) | 0-5 | 0.83 (0.65-1.05) |
| 6 | 0.98 (0.94-1.01) | 0-6 | 0.81(0.63-1.03) |
| 7 | 0.98 (0.95-1.02) | 0-7 | 0.80 (0.61-1.04) |
| 8 | 0.99 (0.95-1.02) | 0-8 | 0.78 (0.60-1.03) |
| 9 | 0.99 (0.96-1.02) | 0-9 | 0.78 (0.58-1.03) |
| 10 | 0.99 (0.96-1.02) | 0-10 | 0.77(0.57-1.03) |
| 11 | 0.99 (0.97-1.02) | 0-11 | 0.76(0.56,1.04) |
| 12 | 0.99(0.97-1.02) | 0-12 | 0.76 (0.56-1.04) |
| 13 | 0.99 (0.96 -1.03) | 0-13 | 0.75(0.55-1.04) |
| 14 | 0.99 (1.02-1.03) | 0-14 | 0.75(0.54-1.04) |
| 15 | 0.99 (0.95-1.02) | 0-15 | 0.74(0.53-1.03) |
| 16 | 0.98 (0.95-1.01) | 0-16 | 0.72(0.51-1.02) |
| 17 | 0.97 (0.94-1.00) | 0-17 | 0.70 (0.50-1.00) |
| 18 | 0.96(0.94-1.00) | 0-18 | 0.68(0.47-0.97) * |
| 19 | 0.95 (0.90-1.00) | 0-19 | 0.64(0.45-0.92) * |
| 20 | 0.93(0.86-1.00) | 0-20 | 0.60(0.41-0.87) * |

*:*P*≤0.05，Single-factor model was adjusted for T5, RF, RH, and SSD.

Abbreviations: RF, rainfall; RH, relative humidity; SSD, sunshine duration; T5, daily average 5 cm ground temperature.

**Table S3** The association between an increase of every 10 µg/m³ of O_3_ and mushroom

poisoning in Guizhou Province from 2019 to 2023.

| **Single-day** | **Relative risk**  **(95%, confidence interval)** | **Multi-day** | **Relative risk**  **(95%, confidence interval)** |
| --- | --- | --- | --- |
| 0 | 0.97(0.90-1.03) | 0-0 | 0.97(0.90-1.03) |
| 1 | 0.97(0.94-0.99) * | 0-1 | 0.93(0.86-1.03) |
| 2 | 0.97 (0.95-0.99) * | 0-2 | 0.90 (0.83-0.98) * |
| 3 | 0.97 (0.96-0.99) * | 0-3 | 0.88 (0.81-0.96) * |
| 4 | 0.98 (0.97-0.99) * | 0-4 | 0.86 (0.79-0.94) * |
| 5 | 0.98 (0.97-0.99) * | 0-5 | 0.85 (0.78-0.92) * |
| 6 | 0.99 (0.97-1.00) | 0-6 | 0.84 (0.76,0.91) * |
| 7 | 0.99 (0.98-1.00) | 0-7 | 0.83 (0.75,0.91) * |
| 8 | 0.99 (0.98-1.00) | 0-8 | 0.82 (0.75,0.90) * |
| 9 | 0.99 (0.98-1.00) | 0-9 | 0.82 (0.74,0.90) * |
| 10 | 1.00 (0.99-1.00) | 0-10 | 0.81 (0.73,0.90) * |
| 11 | 1.00 (0.99-1.01) | 0-11 | 0.81 (0.73,0.90) * |
| 12 | 1.00 (0.99-1.01) | 0-12 | 0.81 (0.73-0.90) * |
| 13 | 1.00 (0.99-1.01) | 0-13 | 0.81 (0.72-0.90) * |
| 14 | 1.00 (0.99-1.01) | 0-14 | 0.80 (0.72-0.90) * |
| 15 | 1.00 (0.98-1.01) | 0-15 | 0.80 (0.71-0.90) * |
| 16 | 0.99 (0.98-1.00) | 0-16 | 0.80 (0.70-0.90) * |
| 17 | 0.99 (0.98-1.00) | 0-17 | 0.79 (0.70-0.89) * |
| 18 | 0.99 (0.98-1.00) | 0-18 | 0.78 (0.69-0.89) * |
| 19 | 0.99 (0.97-1.00) | 0-19 | 0.77(0.67-0.88) * |
| 20 | 0.98 (0.95-1.01) | 0-20 | 0.75 (0.66-0.86) * |

*:*P*≤0.05, Single-factor model was adjusted for T5, RF, RH, and SSD.

Abbreviations: RF, rainfall; RH, relative humidity; SSD, sunshine duration; T5, daily average 5 cm ground temperature.

**Table S4** The association between an increase of every 10 µg/m³ of PM_2.5_ and mushroom poisoning in Guizhou Province from 2019 to 2023.

| **Single-day** | **Relative risk**  **(95%, confidence interval)** | **Multi-day** | **Relative risk**  **(95%, confidence interval)** |
| --- | --- | --- | --- |
| 0 | 0.87(0.73-1.02) | 0-0 | 0.87(0.73-1.02) |
| 1 | 0.93(0.88-0.99) * | 0-1 | 0.81 (0.67-0.98) * |
| 2 | 0.94(0.89-1.00) | 0-2 | 0.76 (0.63-0.92) * |
| 3 | 0.92(0.88-0.97) * | 0-3 | 0.71 (0.58-0.86) * |
| 4 | 0.91(0.88-0.94) * | 0-4 | 0.64 (0.52-0.79) * |
| 5 | 0.9(0.88-0.93) * | 0-5 | 0.58 (0.47-0.72) * |
| 6 | 0.9(0.87-0.93) * | 0-6 | 0.52 (0.42-0.65) * |
| 7 | 0.9(0.87-0.93) * | 0-7 | 0.47 (0.37-0.59) * |
| 8 | 0.91(0.88-0.94) * | 0-8 | 0.43 (0.33-0.54) * |
| 9 | 0.92(0.89-0.94) * | 0-9 | 0.39 (0.30-0.50) * |
| 10 | 0.93(0.91-0.95) * | 0-10 | 0.36 (0.28-0.47) * |
| 11 | 0.94(0.92-0.97) * | 0-11 | 0.34 (0.26-0.45) * |
| 12 | 0.96(0.93-0.98) * | 0-12 | 0.33 (0.25-0.43) * |
| 13 | 0.97(0.94-0.99) * | 0-13 | 0.32 (0.24-0.42) * |
| 14 | 0.98(0.95-1.01) | 0-14 | 0.31(0.23-0.42) * |
| 15 | 0.99(0.96-1.02) | 0-15 | 0.31(0.23-0.41) * |
| 16 | 0.99(0.97-1.02) | 0-16 | 0.31 (0.23-0.41) * |
| 17 | 0.99(0.97-1.02) | 0-17 | 0.30 (0.22-0.41) * |
| 18 | 0.99(0.96-1.01) | 0-18 | 0.30 (0.22-0.40) * |
| 19 | 0.97(0.93-1.01) | 0-19 | 0.29 (0.21-0.39) * |
| 20 | 0.95(0.89-1.02) | 0-20 | 0.28 (0.20-0.38) * |

*:*P*≤0.05, Single-factor model was adjusted for T5, RF, RH, and SSD.

Abbreviations: RF, rainfall; RH, relative humidity; SSD, sunshine duration; T5, daily average 5 cm ground temperature; PM2.5, Particulate Matter 2.5.

**Table S5** The association between an increase of every 10 µg/m³ of SO_2_ and mushroom poisoning in Guizhou Province from 2019 to 2023.

| **Single-day** | **Relative risk**  **(95%, confidence interval)** | **Multi-day** | **Relative risk**  **(95%, confidence interval)** |
| --- | --- | --- | --- |
| 0 | 1.30(0.63-2.70) | 0-0 | 1.30 (0.63-2.70) |
| 1 | 0.79 (0.55-1.13) | 0-1 | 1.03 (0.46-2.30) |
| 2 | 0.69 (0.51-0.94) * | 0-2 | 0.71 (0.29-1.77) |
| 3 | 0.66 (0.53-0.82) * | 0-3 | 0.47 (0.17-1.31) |
| 4 | 0.65 (0.54-0.77) * | 0-4 | 0.30 (0.10-0.92) * |
| 5 | 0.65 (0.54-0.77) * | 0-5 | 0.20 (0.06-0.65) * |
| 6 | 0.66 (0.55-0.79) * | 0-6 | 0.13(0.04-0.46) * |
| 7 | 0.69 (0.58-0.82) * | 0-7 | 0.09 (0.02-0.34) * |
| 8 | 0.72 (0.61-0.85) * | 0-8 | 0.06(0.02-0.27) * |
| 9 | 0.76 (0.66-0.89) * | 0-9 | 0.05 (0.01-0.22) * |
| 10 | 0.81 (0.71-0.93) * | 0-10 | 0.04 (0.01-0.19) * |
| 11 | 0.86 (0.75-0.98) * | 0-11 | 0.03 (0.01-0.18) * |
| 12 | 0.91 (0.79-1.04) | 0-12 | 0.03 (0.01-0.17) * |
| 13 | 0.95 (0.82-1.10) | 0-13 | 0.03 (0.01-0.17) * |
| 14 | 0.98 (0.84-1.14) | 0-14 | 0.03 (0.00-0.17) * |
| 15 | 1.00 (0.86-1.16) | 0-15 | 0.03 (0.00-0.18) * |
| 16 | 0.99 (0.86-1.14) | 0-16 | 0.03 (0.00-0.19) * |
| 17 | 0.96(0.85-1.09) | 0-17 | 0.03 (0.00-0.19) * |
| 18 | 0.91 (0.80-1.04) | 0-18 | 0.03 (0.00-0.18) * |
| 19 | 0.83 (0.67-1.03) | 0-19 | 0.02 (0.00-0.15) * |
| 20 | 0.73(0.52-1.04) | 0-20 | 0.02 (0.00-0.11) * |

*:*P*≤0.05, Single-factor model was adjusted for T5, RF, RH, and SSD.

Abbreviations: RF, rainfall; RH, relative humidity; SSD, sunshine duration; T5, daily average 5 cm ground temperature.
